# On the integrative taxonomy of *Trichophoromyia* Barretto, 1962 and its relationship with *Nyssomyia* Barretto, 1962 (Diptera, Psychodidae, Phlebotominae): species delimitation, phylogeny, genus and subgenus description

**DOI:** 10.1186/s13071-025-07155-6

**Published:** 2025-12-19

**Authors:** Bruno Leite Rodrigues, Thiago Vasconcelos dos Santos, Andreia Fernandes Brilhante, Israel de Souza Pinto, Elisene Gonçala Rocha, Antonio Marques Pereira Júnior, Glaucilene da Silva Costa, Kamila Pereira de França, Keison de Souza Cavalcante, Paloma Helena Fernandes Shimabukuro, Jansen Fernandes Medeiros, Eunice Aparecida Bianchi Galati

**Affiliations:** 1 Faculdade de Saúde Pública (FSP), Universidade de São Paulo (USP), São Paulo, SP Brazil; 2 Seção de Parasitologia, Instituto Evandro Chagas (IEC), Ananindeua, PA Brazil; 3 Centro de Ciências da Saúde E Do Desporto, Universidade Federal Do Acre (UFAC), Rio Branco, AC Brazil; 4 Instituto Federal de Educação, Ciência e Tecnologia Do Espírito Santo (IFES), Ibatiba, ES Brazil; 5 Secretaria de Estado de Educação Do Pará (SEDUC), Novo Progresso, PA Brazil; 6 Laboratório de Análise e Visualização de Dados de Saúde Pública, Fiocruz Rondônia, Porto Velho, RO Brazil; 7 Laboratório Central de Saúde Pública de Rondônia (LACEN–RO), Porto Velho, RO Brazil; 8 Laboratório de Entomologia, Fiocruz Rondônia, Porto Velho, RO Brasil; 9 Vice-Presidência de Pesquisa E Coleções Biológicas, Fundação Oswaldo Cruz, Biobanco da Biodiversidade E Saúde da Fiocruz, Rio de Janeiro, RJ Brazil

**Keywords:** Phylogeny, Molecular systematics, DNA barcoding, Multilocus, Multispecies coalescent, Species delimitation

## Abstract

**Background:**

Thes and fly genus *Trichophoromyia* Barretto, 1962 is one of the most diverse in the subfamily Phlebotominae. The taxonomy and systematics of this group is complex due to both a high similarity among species and unclear relationships among other sand fly groups within the subtribe Psychodopygina Galati, 1995. Despite their great relevance as vectors of *Leishmania* spp., few studies have explored the usefulness of molecular markers in studying the diversity of this group.

**Methods:**

Here, we evaluated the use of barcode sequences of the cytochrome coxidasesubunit I gene (*COI*) for identifying several *Trichophoromyia* spp., by inferring intra- and interspecific genetic distances, in addition to performing a set of several single-locus species delimitation approaches using discovery methods. Moreover, we employed a multilocus dataset of four independent molecular markers (*COI, ITS2, 28S and PARA*) to infer the phylogenetic species tree, estimate divergence times and delimit species under a validation model.

**Results:**

The phylogenetic inferences confirmed the paraphyly of *Trichophoromyia* and *Nyssomyia* Barretto, 1962. Thus, two new genera, named *Reburrus* gen. nov. and *Shawmyia* gen. nov., were proposed to accommodate sand fly species that did not fit in the aforementioned groups. Additionally, a new sub genus was proposed: *Trichophoromyia* (*Dilermandomyia*) subg. nov., containing most species of *Trichophoromyia*. A recent speciation history was also estimated, with most of the species studied diversifying during the Pleistocene. However, our dataset was insufficient to fully resolve relationships within *Trichophoromyia* (*Dilermandomyia*) subg. nov. Many species showed paraphyletic patterns in the gene trees, and some could not be reliably identified and delimited using both *COI* barcodes and multilocus tools.

**Conclusions:**

The sand fly genus *Trichophoromyia* exhibits a complex diversification history. Our phylogenetic inference and morphological observations of *Nyssomyia* and *Trichophoromyia*, allowed us to propose new groups for the Psychodopygina subtribe. However, the prevalence of species-level paraphyletic patterns for *Trichophoromyia* (*Dilermandomyia*) subg.nov., showed that further assessment of this group requires a broader locus sampling combined with detailed morphological analysis.

**Graphical Abstract:**

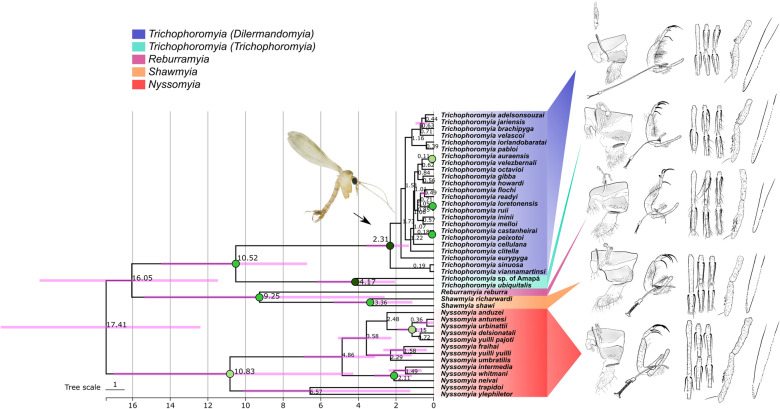

**Supplementary Information:**

The online version contains supplementary material available at 10.1186/s13071-025-07155-6.

## Background

The Neotropical sand fly genus *Trichophoromyia* Barretto, 1962 currently comprises 48 species [1, 2]. Only females are hematophagous, and some of them can act as biological vectors of *Leishmania* parasites that infect humans and other animals [3, 4]. For example, *Trichophoromyia ubiquitalis* (Mangabeira, 1942) has been considered to be the main vector of *Leishmania* (*Viannia*) *lainsoni* Silveira, Shaw, Braga & Ishikawa, 1987, which is the causal agent of cutaneous leishmaniasis in the Brazilian Amazon [5, 6]. Although males of *Trichophoromyia* can be distinguished among species based on morphological characters, the same is not true for most females [7], posing significant challenges for accurate identification. As the species differ in feeding sources and behavioral patterns, their species-level identification is one of the pillars of studies on vectorial capacity and control of this group.

The classification of the subfamily Phlebotominae has long been debated. However, the most well-founded and widely accepted proposal divides the approximately 550 species of the Neotropical region [8, 9] into two tribes, five subtribes and 23 genera [7, 10]. Of these, *Trichophoromyia* is allocated in the subtribe Psychodopygina. Phylogenetic analysis of morphological characteristics suggests that this genus should be classified alongside *Nyssomyia* Barretto, 1962, as one of the most derived groups. However, for both genera, the phylogenetic positioning of its species appears to be an emerging problem due to the paraphyletic status of some taxa based on molecular traits [11]. Therefore, studies evaluating the species delimitation and phylogeny of this group are important for a better understanding of sand fly diversity, allowing a proper natural classification.

Integrative taxonomic approaches have been applied to sand flies and have yielded significant improvements in phylogenetic reconstructions, molecular identification, detection of cryptic diversity and association between sexes [12, 13]. However, *Trichophoromyia* remains one of the most neglected genus regarding this type of approach, with only 20% of its species sequenced for any molecular marker until very recently [13]. Nevertheless, a fragment of the cytochrome* c* oxidase subunit I gene (*COI*) is now being used to validate species descriptions [14, 15]. In addition, interesting results from studies on some closely related species have revealed discordant patterns, with some nominal species split into multiple evolutionary lineages, whereas others merged with morphologically distinct taxa [16]. These results show that molecular markers are useful for improving current understanding of the diversity of *Trichophoromyia*, but many gaps remain to be filled.

The objective of this study was to evaluate a comprehensive sampling of *COI* gene fragments for the identification of sand flies belonging to the genus *Trichophoromyia*. In addition, a multilocus dataset encompassing both *COI* and nuclear gene fragments was employed to verify the phylogenetic positioning of the sampled species, particularly in relation to the genus *Nyssomyia*. Finally, the dataset was utilized to estimate the divergence times of the recovered clades and to implement a species delimitation method under the multispecies coalescent model.

## Methods

### Sampling and processing

**Fig. 1:**
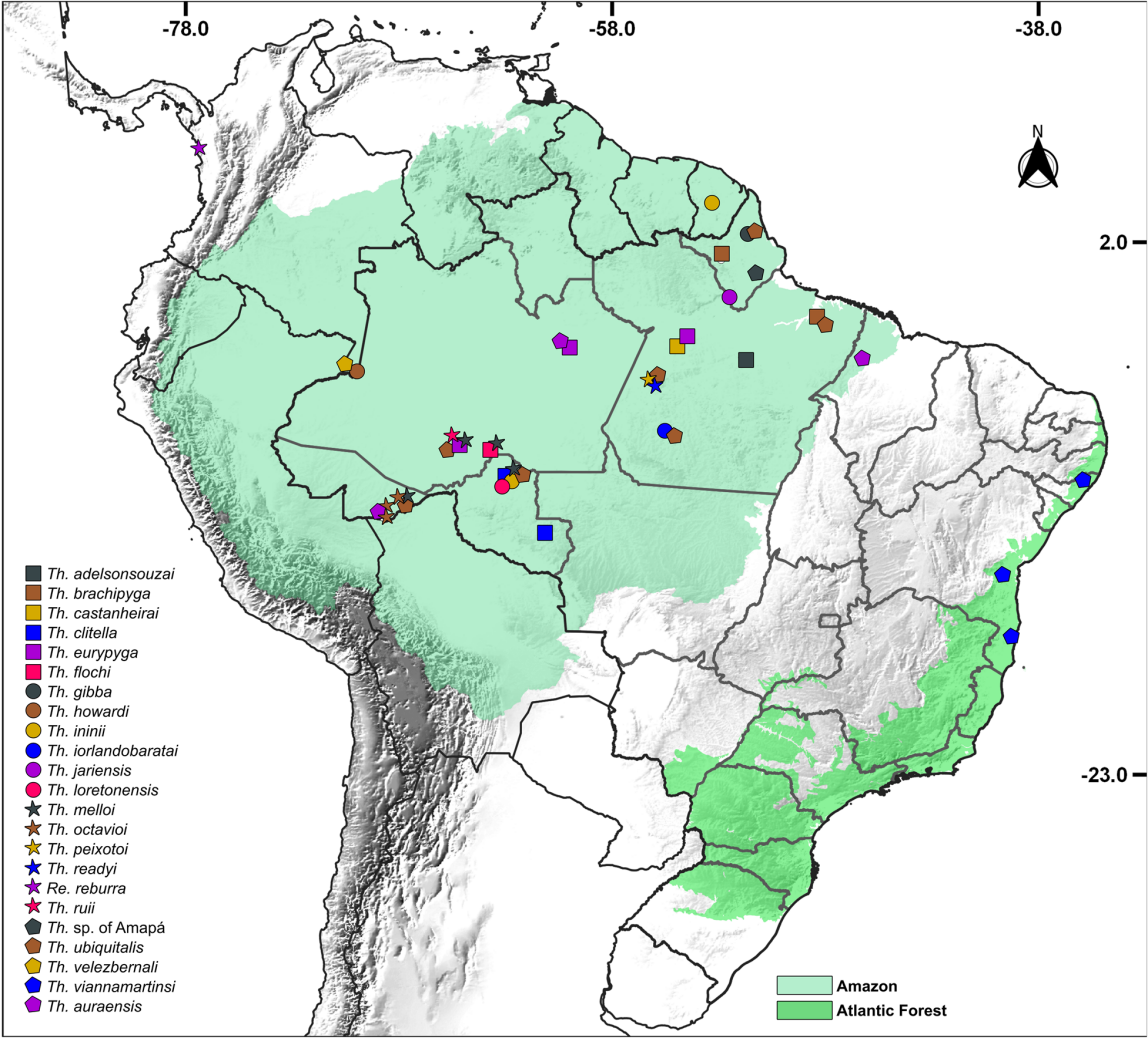
Geographical distribution of *Trichophoromyia* specimens analyzed in this study (both those processed here and those extracted from GenBank). The overlapping symbols correspond to different species from the same geographical location. *Th*.,* Trichophoromyia*

Entomological collections were performed between 2014 and 2024 in 17 municipalities of eight Brazilian states (see Additional file 1: Table S1). U.S. Centers for Disease Control and Prevention (CDC)-type light traps were installed overnight in fragments of the Amazon Rainforest (states of Acre, Amapá, Amazonas, Pará and Rondônia) and Atlantic Forest (states of Alagoas and Bahia) biomes (Fig. 1). All sand flies used for molecular procedures were preserved in ethanol (70–95%) at − 20 °C.

Following a screening process, the sand flies were dissected for mounting on slides and to separate tissue for DNA extraction. The legs of the sand fly were prioritized for DNA extraction due to their minimal taxonomic relevance for the analyzed groups. This approach enabled the head, thorax and abdomen of each specimen to be mounted on a slide. However, there is a high probability that legs will be lost due to the fragility of this structure. Consequently, for specimens that exhibited three or more legs following the initial trial, the legs were dissected and stored in sterile microtubes; for those sand fly specimens with fewer legs, the thorax was dissected and stored for subsequent DNA extraction. This approach guaranteed the availability of sufficient tissue for DNA extraction, according to the protocol employed in this study. Following the dissection process, the specimens were prepared for slide mounting according to the methodology outlined by Rodrigues et al. [17].

For the morphological studies, in addition to the freshly collected specimens used in the molecular analysis, we extracted *Trichophoromyia* and *Nyssomyia* sand flies deposited in the reference collections of the Departamento de Epidemiologia, Faculdade de Saúde Pública, Universidade de São Paulo (HEP/FSP/USP), Museu de Zoologia da Universidade de São Paulo (MZUSP) and the Coleção de Flebotomíneos do Instituto René Rachou (Fiocruz/COLFLEB). The list of the analyzed taxa for morphological inferences and their localities is available in Additional file 1: Table S2.

Sand flies were morphologically identified using the dichotomous keys of Galati [7] as the basis, and the original descriptions of nearly all *Trichophoromyia* spp. analyzed in this study were consulted.

Images of specimens were captured with an AxioCam camera model ‘105 color’ (Carl Zeiss MicroImaging GmbH, Jena, Germany) coupled to a light microscope. The illustrations were initially executed in graphite, employing an Olympus camera lucida tool (Olympus Corp., Tokyo, Japan) affixed to a microscope, and then digitally finalized using Procreate v5.3 (Savage Interactive, Hobart, Australia) on an iPad (Apple Inc., Cupertino, CA, USA).

### DNA extraction, PCR and sequencing

The total DNA extraction process was carried out for each collected sand fly specimen individually. This procedure was performed using the residual tissue from the clarification and slide-mounting methodologies. The extraction method employed was a salt-based protocol using Digsol Buffer (ethylenediaminetetraacetic acid [EDTA] 20 mM; Tris–HCl 50 mM; NaCl 117 mM; sodium dodecyl sulfate [SDS] 1%), as described by Rodrigues et al. [16].

DNA amplification targeted four fragments of independent molecular markers: (i) *COI* from mitochondrial DNA (mtDNA); (ii) nuclear (nDNA) internal transcribed spacer 2 (*ITS2*); (iii) domains D1-D2 from the large ribosomal subunit RNA gene (*28S* rDNA); and (iv) the paralytic (*PARA*) gene.

The analyzed fragment of *COI* has been used as the primary marker for the molecular identification of animals through DNA barcoding initiatives [18]. This approach has proven to be highly effective for delimiting sand fly species and detecting cryptic diversity [13]. The *28S* rDNA is a conserved DNA fragment that is typically employed for phylogenetic inferences [11, 19]. In contrast, the *ITS2* marker is utilized for closely-related and population-level studies [15, 20–22]. Finally, the *PARA* gene has been implicated in the regulation of copulatory courtship songs in *Drosophila* [23, 24], and it was previous employed to analyze the population genetic structure of the *Lutzomyia longipalpis* complex [25].

PCR analyses were carried out in accordance with the manufacturer's instructions using the GoTaq™ Green Master Mix (Promega, Madison, WI, USA) in a final reaction volume of 25 μl (23 μl of PCR Mix + 2 μl of DNA template), with a final primer concentration of 0.2 mM. In cases of over-amplification or nonspecific bands, a second round of PCR using 1 μl of DNA template was performed for* ITS2*. The pairs of primers utilized for each molecular fragment in this procedure were: *COI* (LCO1490 [5′-GGTCAACAAATCATAAAGATATTGG-3′]; HCO2198 [5′-TAAACTTCAGGGTGACCAAAAAATCA-3′]) [26]; *28S* (C1’ [5′-ACCCGCTGAATTTAAGCAT-3′]; D2 [5′-TCCGTGTTTCAAGACGGG-3′]) [27]; *ITS2* (C1A [5′-CCTGCTTAGTTTCTTTTCCTCCGCT-3′]; JTS3 [5′-CGCAGCTAACTGTGTGAAATC-3′]) [20]; and *PARA* (5llpara2 [5′-ACGGACTTCATGCATTCATTC-3′]; 3llpara1 [5′-TGGTGCTGATAAACTTGACG-3′]) [23]. All PCR cycling regimens consisted of an initial denaturation at 95 °C for 2 min and a final extension of 72 °C for 10 min, with 35 repetitive cycles between these two phases which were set as follows: *COI* (95 °C for 1 min, 54 °C for 1 min and 72 °C for 1 min 30 s), *28S* (95 °C for 1 min, 58 °C for 1 min and 72 °C for 1 min), *ITS2* (95 °C for 30 s, 62 °C for 1 min and 72 °C for 1 min) and *PARA* (95 °C for 30 s, 60 °C for 30 s and 72 °C for 30 s).

Samples were checked via electrophoresis in 1% agarose gels stained with GelRed (Biotium Inc., Fremont, CA, USA), and all positive reactions with the expected molecular size were sent to ACTGene Análises Moleculares (Brazil) for the purification and sequencing of the PCR products in both directions (forward and reverse) using the ABI-Prism 3500 Genetic Analyzer (Applied Biosystems, Thermo Fisher Scientific, Waltham, MA, USA).

### Dataset assembly and sequence analysis

The electropherograms were manually inspected to trim primer sequences and to assemble consensus sequences using SeqTrace v.0.9, and the resulting sequences were subsequently deposited in the NCBI GenBank database [28], where they were assigned accession numbers for the following genes: *COI* (*n* = 152; PV624881–PV625032), *28S* (*n* = 29; PV628406–PV628434), *ITS2* (*n* = 24; PV636952–PV636975) and *PARA* (*n* = 28; PV651723–PV651750).

In order to enhance the quality of the analyzed dataset, we performed a data integration approach by incorporating publicly available sequences of *Trichophoromyia* spp. from the NCBI GenBank into our existing dataset. This was complemented by the inclusion of sequences from *Nyssomyia* spp., given the documented close relatedness between these two genera [7] and the apparent paraphyletic pattern across these two groups [11]. To this end, the reliability of the morphological identification, number of species and the fragment size were prioritized. Up to five sequences were selected in cases of species with numerous available GenBank accessions; in cases where *COI* barcode fragments were partial, only those exceeding 600 bp were selected for the analyses. The complete analyzed dataset for each gene fragment is available in Additional file 1: Table S1.

### Phylogenetic inference—*COI* gene trees

Maximum-likelihood analysis was performed using the IQ-TREE 2 software [29]. The optimal partition scheme (considering the three codon positions) and the substitution model were obtained through the utilization of the −MF+MERGE option, employing the PartitionFinder [30] and ModelFinder methods [31], respectively. These methods are directly implemented in IQ-TREE. Subsequently, the analysis was performed with 10,000 rounds of ultra-fast bootstrap [32] and 1000 SH-like approximate likelihood ratio test [33] to evaluate branch support. In this study, we defined “confident clades” as those with SH-aLRT values > 80% and UFBoot values > 95%. Due to the relatedness of the Lutzomyiina subtribe in relation to Psychodopygina, three sequences of *Lutzomyia longipalpis* (Lutz & Neiva, 1912) (KP112580–82) were included as outgroups for *COI* analyses to root the tree.

Bayesian Inference (BI) analysis was performed using BEAST v2 [34]. To this end, we implemented strict clock and Yule priors, assuming a constant-like speciation rate due to the recent divergence hypothesis of *Trichophoromyia* and *Nyssomyia*. For model selection, we implemented the bModelTest plugin, to average over the evolutionary substitution models during gene tree reconstruction [35]. Two runs were performed independently for 15,000,000 generations (sampling ‘.log’ and ‘.tree’ files every 1000). The trace logs were then subjected to visual analysis in Tracer v.1.7 to ascertain the convergence and effective sample size (ESS) values, all of which exceed 200. The tree files from both runs were merged using LogCombiner v2.5.2 with a 10% burn-in period for each, and then a maximum clade credibility (MCC) tree was generated from the retained trees in TreeAnnotator v2.6.6. The programs FigTree v.1.4.4, Inkscape v1.4 and tvBOT [36] were used to visualize and edit the trees.

### Molecular species identification and delimitation—*COI* dataset

Pairwise genetic distances were generated for both intra- and interspecific comparisons using MEGA v7.

To assess the utility of *COI* barcodes in identifying different nominal species within our *COI* dataset, we performed a sequence-similarity analysis using the Best Match (BM) and Best Close Match (BCM) criteria implemented in TaxonDNA [37]. In this case, successful identification requires that a query sequence has the closest barcode match (estimated using uncorrected *p* distances) with a sequence from the same nominal species. Conversely, the identification is considered ambiguous or incorrect if the former has the closest matches with barcodes of different species [37]. For this study, a dataset was compiled that encompassed all *Trichophoromyia* and *Nyssomyia COI* sequences that had been identified at the species level. This comprehensive dataset comprised a total of 247 sequences, not filtering identical haplotypes, with each sequence serving as the query sequence and being compared to the remaining 246 sequences, which constituted the reference library.

Furthermore, to investigate the potential of various species partition schemes for *COI* barcode datasets, a series of single-locus species delimitation algorithms (discovery methods) were implemented for *Trichophoromyia* and *Nyssomyia*. In this case, taxa were assigned to groups without a priori information, being identified as different molecular operational taxonomic units (MOTUs) and compared to the morphological identification of samples. These algorithms are predicated on divergent assumptions for the partitioning of data into hypothetical groups, and they were implemented with varied parameter settings to ascertain the degree of agreement between them and the analyzed nominal species of this study. All species delimitation analyses were performed using the full *COI* dataset of 254 sequences of *Trichophoromyia* and *Nyssomyia* (Additional file 1: Table S1), also including those identified at the genus level.

Here, we considered the delimitations retrieved from the following algorithms: the distance-based Automatic Barcode Gap Discovery (ABGD [38]), Assemble Species by Automatic Partitioning (ASAP [39]) and Refined Single Linkage (RESL [40]) algorithms, in addition to the tree-based Generalized Mixed Yule Coalescent (GMYC [41]) and Poisson Tree Processes (PTP [42]) algorithms.

The ABGD algorithm tries to identify the barcode gap that separates intra- from interspecific distances at a given pairwise distance matrix, generating a hypothetical partition based on the genetic divergence of samples. For ABGD, the analyses were run in the webserver (https://bioinfo.mnhn.fr/abi/public/abgd/, accessed 14 April 2025) using both *p* distances and the Kimura 2-parameter (K2P) model to estimate the pairwise distance matrix, with the following parameters: *P*min = 0.005, *P*max = 0.1 and *X* = 1.0. We considered the recursive partitions with prior maximal distance of *P* = 0.009729 and *P* = 0.013572 as they provided different partition schemes.

The ASAP method, which is similar to the ABGD method, is also a distance-based algorithm that employs threshold values to distinguish between intra- and interspecific variation. We performed the analyses of ASAP in its webserver (https://bioinfo.mnhn.fr/abi/public/asap/asapold.html, accessed 14 April 2025), also using uncorrected *p* distances and K2P models. We considered the best ASAP partition to be the one with the lowest ‘asap-score,’ with a threshold distance of at least 0.5% to avoid overestimated scenarios.

The RESL method employs single linkage clustering as a tool for the preliminary assignment of the specimens into MOTUs. In this study, *COI* sequences were initially clustered by employing a fixed 2.2% threshold of uncorrected *p* distance and then refined into the final partitions by Markov clustering. The RESL clusters of our sequences were accessed directly in the BOLD Systems environment using the ‘cluster sequences’ tool inside the *COI* dataset page.

Lastly, we performed two tree-based approaches: PTP and GMYC. These two algorithms are coalescent-based methods that seek to identify transition points between species-level processes (speciation) and population-level processes (allele coalescence) events in phylogenetic trees. The difference between the PTP and GMYC methods is the input, with GMYC designed to perform with ultrametric gene trees, while PTP does not require that the input tree be time-calibrated. The analyses of both algorithms were run in their respective webservers (https://mptp.h-its.org/ and https://species.h-its.org/gmyc/, accessed 14 April 2025). We used the maximum likelihood (ML) phylogenetic gene tree by IQ-TREE and the Bayesian MCC as input to perform PTP and GMYC delimitations, respectively. Both algorithms were run using single and multiple thresholds.

### Species tree and validation under the multispecies coalescent model

The multilocus dataset was employed to infer the species tree and species delimitation under the multispecies coalescent (MSC) model [43]. This model naturally accommodates gene tree fluctuations across the genome and potential gene tree versus species-tree discordance. MSC-based inferences have proven to be useful for phylogenetic inferences based on the rapid succession of speciation events [44, 45]. In addition to tree topology and branch supports, the divergence times of clades were estimated using the StarBeast2 template [46], which is included in the BEAST v2 software. For this analysis, the following priors were assumed: (i) strict clock; (ii) Yule model; and (iii) analytical population size integration. The estimation of site models was conducted for each partition, as previously outlined for the *COI*-only BEAST analysis. The substitution rate of *COI* partition was set at 0.0115 mutations per nucleotide per million years, as described by Brower [47]. The remaining nDNA markers were estimated in the analysis relative to the mtDNA rate. Two analyses were executed concurrently for 100,000,000 steps, with a sampling frequency of 5000. It was observed that all parameters attained ESS values > 200.

In order to perform multilocus species delimitation under the MSC model, we employed the Bayesian Phylogenetics and Phylogeography (BPP) v4.6 software [48] to assess the relative robustness of various candidate species delimitation hypotheses. This was achieved through the implementation of the fixed guide tree analysis (A10) [49, 50] and the joint species delimitation and species tree estimation (A11) [51]. The coalescent-based analysis of BPP has been demonstrated to outperform other discovery approaches [52]. It has also been shown to be reliable in delimiting recently diverged taxa with paraphyletic patterns in gene trees, and with some degree of gene flow across lineages. This approach, termed “validation methods,” for species delimitation [53] employs a priori knowledge on the partitioning of the dataset, assigning species to groups. Thereafter, the reversible-jump Markov Chain Monte Carlo (rjMCMC) algorithm transitions between various delimitation models by collapsing nodes throughout the species tree, thereby calculating the posterior distribution of speciation probabilities for each split in the guide tree. Here, BPP analyses were conducted on a dataset comprising only specimens identified at the species level of the subgenus *Trichophoromyia* (*Dilermandomyia*) subg. nov., as the dataset exhibited significant discordance between morphological identification and the formation of clades with reciprocal monophyly in gene tree topologies (see Results section). *Trichophoromyia* (*Trichophoromyia*) *s. str.* was utilized as the outgroup, bringing the final dataset to 27 *Trichophoromyia* species, and 185, 33, 33 and 26 sequences of *COI*, *28S*, *ITS2* and *PARA*, respectively. The priors for the population size (*θ*) and species divergence times (*τ*) do not have default values, and a different combination was tested to check the consistency of the results [54, 55]. All analyses were executed for 200,000 generations, with a sampling interval of every two generations, and a 20% burn-in phase. The guide tree was based on the topology of the multilocus species tree generated by StarBeast2. Each analysis was performed in duplicate, ensuring the reliability of the results.

## Results

### Taxonomy: proposals of a new subgenus and two genera

An examination of the species of *Trichophoromyia* yielded substantial disparities in both morphology and molecular characteristics, suggesting a subdivision of this genus into two subgenera: *Trichophoromyia* (*Trichophoromyia*) *s. str.* and *Trichophoromyia* (*Dilermandomyia*) subg. nov., predicated on monophyletic clades derived from molecular analyses and morphological discrepancies in male terminalia and female spermathecae. However, our analysis indicated that one of the *Trichophoromyia* species was not adequately allocated to any of the proposed subgenera, both by morphological and molecular characteristics, resulting in the proposal of the monotypic genus *Reburramyia* gen. nov. In addition, the molecular phylogeny also showed the paraphyletic status of *Nyssomyia* spp., due to the positioning of two species, for which morphological discrepancies among them and *Nyssomyia s. str*. allowed us to propose the new sand fly genus *Shawmyia* gen. nov. All of these new group proposals are based on the monophyletic pattern of our molecular species tree analysis (see Methods section), which included one mtDNA and three nDNA markers. To comply with the regulations set out in Article 8.5 of the amended 2012 version of the International Code of Zoological Nomenclature, details of the new names have been submitted to ZooBank. The Life Science Identifier (LSID) of the article is urn:lsid:zoobank.org:pub:67AAB1C9-BC11-41BD-A98A-2D620544E158.

The *Trichophoromyia* and *Nyssomyia* species that were reassigned to the new proposed genera have intermediate morphological characteristics between the two groups. In this context, all of the genera analyzed possess a set of morphological characters useful for defining the diagnosis of each group, justifying the proposal of the new generic and subgeneric taxa (Table 1).

**Table 1:** Morphological characters of the genera *Nyssomyia*, *Trichophoromyia, Shawmyia* and *Reburramyia*

Sex	Morphological characters	*Nyssomyia*	*Shawmyia*	*Reburramyia*	*Trichophoromyia*
♂/♀	Presence of simple setae on flagellomeres	Few, generally in apical ones (FXII–FXIV)	Few, in all segments (FI–FXIV)	Great abundance, in all segments (FI–FXIV)	Few, variable in FI–FVI, present in FVII–FXIV
	Simple setae in FI	Absent	Few (approx. 2)	Many (> 10)	Absent or few (approx. 1–2)
	Simple setae in FII–V	Absent or few (approx. 1–2)	Few (approx. 2–3)	Many (> 10)	Absent or few (approx. 1–4)
	Newstead's sensilla on palpal segment 2	Absent	Absent	Absent	Generally present
	Newstead's sensilla on palpal segment 3	Grouped in a slightly swollen area near to the middle of P3	Scattered, without swollen area	Scattered, without swollen area	Scattered, without swollen area
♂	Implantation of the internal spine of the gonostyle	In the middle or distal third	In the basal third	In the basal third	In the basal third
	Length of apical spine (gonostyle)	As long as gonostyle	As long as gonostyle	Shorter than gonostyle	Shorter than gonostyle
	Terminalia in relation to the mesonotum length (disregarding the apical spine)	Shorter	Shorter	Longer	Longer
♀	External teeth of the lacinia of the maxilla	Two rows	Two rows	One row	Two rows
	Spermathecae	5–15 rings	Approx. 12–15 rings	Approx. 15–18 rings^a^	25 or more
	Cercus, in lateral view	Long, length ≥ twofold its width	Long, length ≥ twofold its width	Long, length ≥ twofold its width	Short, length < twofold its width

Observations are based on the specimens listed in Additional file 1: Table S2, and are adapted and improved from Zapata et al. [11]

*FI–FXIV* Flagellomere segments,* P1–P5* palpal segments

^a^The basal ones are difficult to delimit due to excrescences

Regarding *Trichophoromyia* subgenera, morphological diagnosis to differentiate these are clearly associated to genitalia aspects: males of *Trichophoromyia* (*Trichophoromyia*) *s. str.* have shorter aedeagal ducts (threefold or less longer than the sperm pump), while *Trichophoromyia* (*Dilermandomyia*) subg. nov. presents longer ducts (usually sixfold longer than the sperm pump). In a similar manner, the spermathecae of the first group exhibit an apical ring that is threefold or less longer than the preapical one, while for the latter group, the apical ring is at least sixfold longer than the preapical one (Table 1 and Table 2).

The differential diagnosis of *Trichophoromyia* and *Reburramyia* gen. nov. can be seen in flagellomeres, lacinia of the maxilla, female spermathecae and cercus. A distinguishing characteristic of *Reburramyia reburra* gen. nov., comb. nov. is the presence of a great abundance of simple setae on all flagellomeres. In contrast, other *Trichophoromyia* spp. exhibit variable presence of these structures, albeit always in small quantities, throughout the flagellomeres. Furthermore, the presence of a single row of external teeth on the lacinia of the maxilla, in contrast to the expected two rows, along with the excrescences on spermathecae ducts and an elongated cercus, supports the differential diagnosis of both genera (Table 1).

Lastly, *Nyssomyia* and *Shawmyia* gen. nov. are different in terms of their flagellomeres, palpi and male terminalia. The first group generally present simple setae only in the apical segments of antennae, while in both species of *Shawmyia* gen. nov. simple setae can be seen in all flagellomeres. Moreover, Newstead's sensilla on P3 is grouped, forming a slightly swollen area near to the middle of the segment in *Nyssomyia*, and it is scattered (without swollen area) in the latter group. Nevertheless, the most evident diagnostic characteristic among these genera is the implantation of the internal spine of the gonostyle, which is isolated in the basal third for *Shawmyia* gen. nov.. This characteristic is very similar to what was observed in *Reburramyia* gen. nov. and *Trichophoromyia*, but not in *Nyssomyia* (Table 1).

Following the recommendation of Marcondes [56], the abbreviation of the two new subgenera of *Trichophoromyia* and the two new genera are “*Th*. (*Thp*.)”, “*Th*. (*Dil*.)”, “*Re*.” and “*Sh*.”, respectively.

Family Psychodidae Newman, 1834

Subfamily Phlebotominae Rondani & Berté, in Rondani 1840

Tribe Phlebotomini Rondani & Berté, 1840

Subtribe Psychodopygina Galati, 1995

**Fig. 2:**
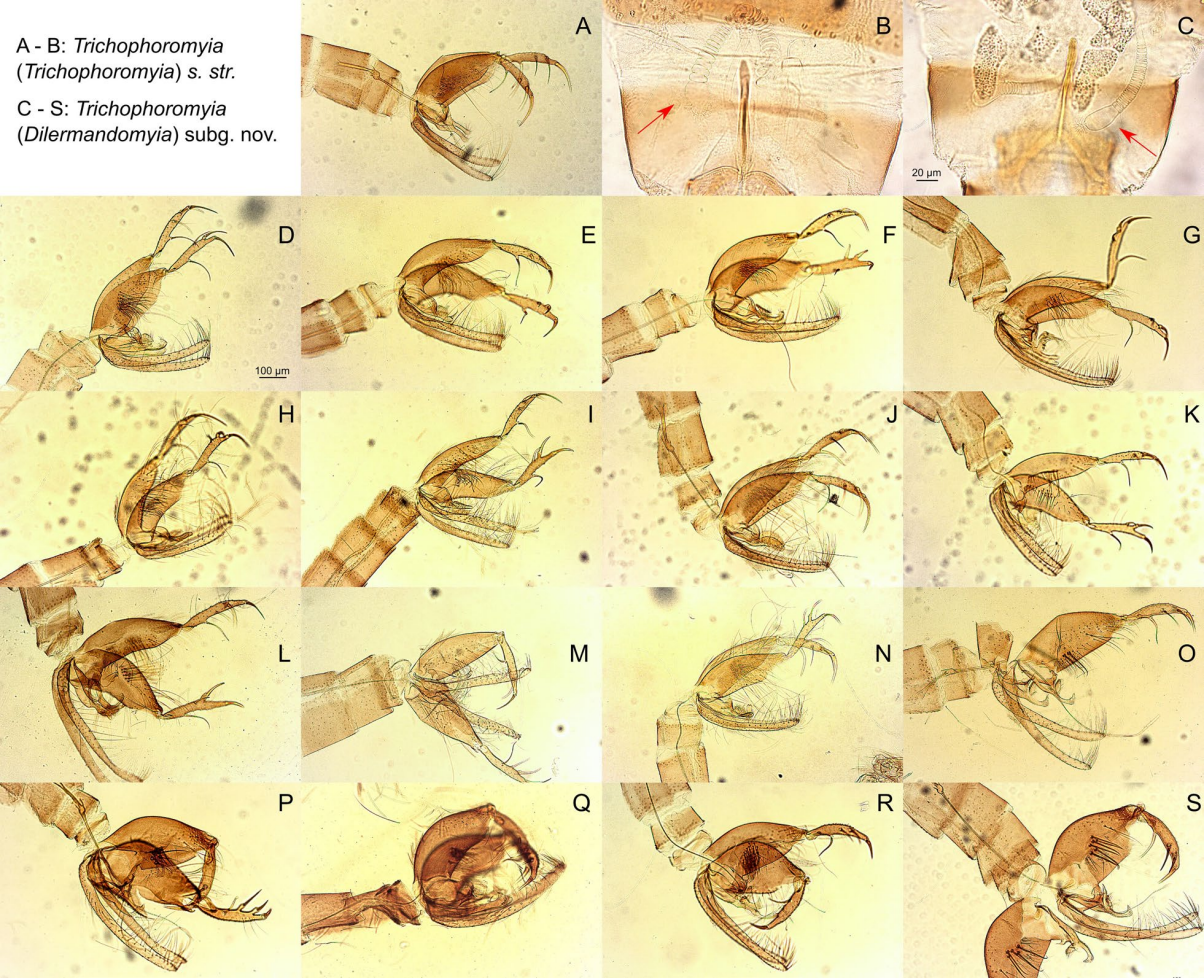
Male genitalia and female spermathecae of *Trichophoromyia* spp. processed and analyzed in this study. **A**, **B** *Th. ubiquitalis*, **C** female of *Trichophoromyia (Dilermandomyia)* subg. nov., **D** *Th. adelsonsouzai*, **E** *Th. readyi*, **F** *Th. peixotoi*, **G** *Th. castanheirai*, **H** *Th. auraensis*, **I** *Th. octavioi*, **J** *Th. ruii*, **K** *Th. melloi*, **L** *Th. iorlandobaratai*, **M** *Th. clitella*, **N** *Th. flochi*, **O** *Th. viannamartinsi*, **P** *Th. gibba*, **Q** *Th. jariensis*, **R** *Th. eurypyga*, **S** *Th. brachipyga*

**Fig. 3:**
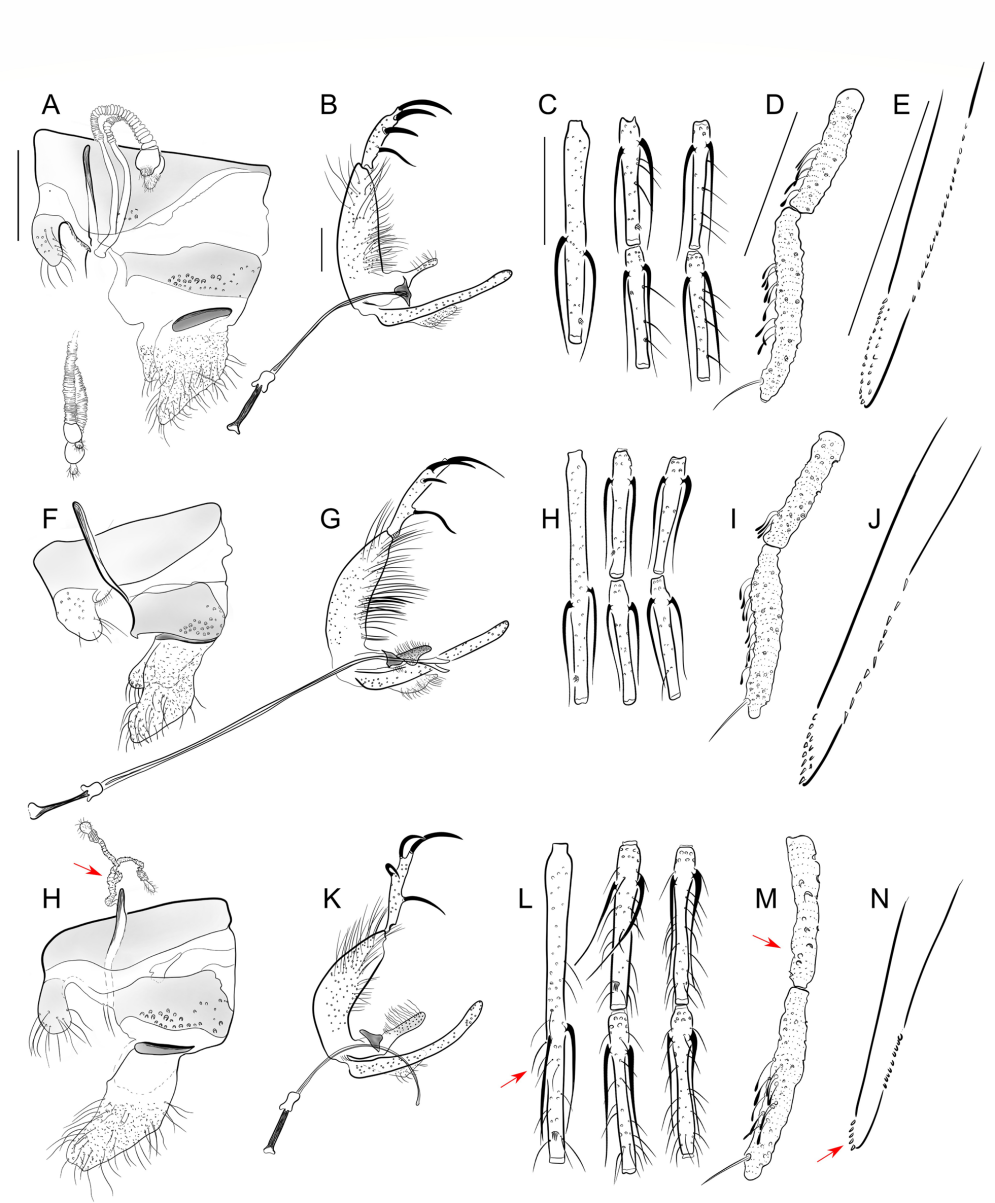
Main morphological characters used to identify sand flies of *Trichophoromyia* (*Trichophoromyia*) (**A**–**E**), *Trichophoromyia* (*Dilermandomyia*) (**F**–**J**), and *Reburramyia* (**H**–**M**). **A**, **F** and **G** Female spermathecae of *Th. ubiquitalis*, *Th. auraensis* and *Re. reburra*, respectively. **B**, **G**, **K ** Male terminalia. **C**, **H**, **L** Male flagellomeres FI to FV. **D**, **I,** **H** Male palpi P2 and P3 with Newstead’s sensilla. **E**, **J**, **N** Female lacinia of the maxilla. Red arrows indicate diagnostic characters for the identification of the genus *Reburramyia*. Different illustrations of the same structure are in the same scale. Scale bars: 100 μm

*Trichophoromyia (Trichophoromyia)* Barretto, 1962 (Figs.  2a, b; Fig. 3a–e)

Type-species: *Phlebotomus ubiquitalis* Mangabeira, 1942.

**Diagnosis**. **Males**: aedeagal ducts threefold or less longer than the sperm pump. **Females**: spermathecae with a terminal knob sessile or spherical, and the terminal ring threefold of less longer than the preapical one.

Composition—five species: *Trichophoromyia meirai* (Causey & Damasceno, 1945), *Trichophoromyia omagua* (Martins, Llanos & Silva, 1976), *Trichophoromyia ubiquitalis* (Mangabeira, 1942), *Trichophoromyia uniniensis* Ladeia-Andrade, Fé, Sanguinette & Andrade Filho, 2014 and *Trichophoromyia* sp. of Amapá (to be formally described).

*Trichophoromyia (Dilermandomyia)* subg. nov. Rodrigues & Galati (Figs. 2c–s, 3f–g)

LSID: urn:lsid:zoobank.org:act:68A810BC-269F-45B4-96D3-87CD6EDC0F46

Type-species: *Phlebotomus auraensis* Mangabeira, 1942.

**Diagnosis**. **Males**: aedeagal ducts fourfold (but usually sixfold) longer than the length of the sperm pump. **Females**: apical ring of spermathecae sixfold longer in relation to the preapical ring.

Composition–43 species: *Trichophoromyia acostai* (Llanos, 1966), *Trichophoromyia adelsonsouzai* Santos, Silva, Barata, Andrade & Galati, 2013, *Trichophoromyia arevaloi* Galati & Cáceres, 1999, *Trichophoromyia auraensis* (Mangabeira, 1942), *Trichophoromyia beniensis* (Le Pont & Desjeux, 1987), *Trichophoromyia bettinii* (Feliciangeli, Ramirez Pérez & Ramirez, 1988), *Trichophoromyia brachipyga* (Mangabeira, 1942), *Trichophoromyia castanheirai* (Damasceno, Causey & Arouck, 1945), *Trichophoromyia cellulana* (Young, 1979), *Trichophoromyia clitella* (Young & Pérez, 1994), *Trichophoromyia dilermandoi* Lopes & Shimabukuro, 2025, *Trichophoromyia dunhami* (Causey & Damasceno, 1945), *Trichophoromyia eurypyga* (Martins, Falcão & Silva, 1963), *Trichophoromyia flochi* (Abonnenc & Chassignet, 1948), *Trichophoromyia gibba* (Young & Arias, 1994), *Trichophoromyia howardi* (Young, 1979), *Trichophoromyia incasica* (Llanos, 1966), *Trichophoromyia ininii* (Floch & Abonnenc, 1943), *Trichophoromyia iorlandobaratai* Vasconcelos dos Santos, Santos Neto, Sánches-Uzcategui & Galardo, 2018, *Trichophoromyia jariensis* Cavalcante, Rodrigues & Galati, 2024, *Trichophoromyia lopesi* (Damasceno, Causey & Arouck, 1945), *Trichophoromyia loretonensis* (Llanos, 1964), *Trichophoromyia macrisae* Méndez-Cardona & Cabrera-Quintero, 2024, *Trichophoromyia melloi* (Causey & Damasceno, 1945), *Trichophoromyia napoensis* (Young & Rogers, 1984), *Trichophoromyia nautaensis* (Fernandez, Lopez, Cardenas & Requena, 2015), *Trichophoromyia nemorosa* (Young & Pérez, 1994), *Trichophoromyia octavioi* (Vargas, 1949), *Trichophoromyia pabloi* (Barreto, Burbano & Young, 2002), *Trichophoromyia pastazaensis* (Fernandez, Carbajal, Alexander & Need, 1993), *Trichophoromyia peixotoi* Rodrigues, Pinto & Galati, 2023, *Trichophoromyia readyi* (Ryan, 1986), *Trichophoromyia reinerti* (Young & Duncan, 1994), *Trichophoromyia rostrans* (Summers, 1912), *Trichophoromyia ruifreitasi* Oliveira, Teles, Medeiros, Camargo & Pessoa, 2015, *Trichophoromyia ruii* (Arias & Young, 1982), *Trichophoromyia saltuosa* (Young, 1979), *Trichophoromyia sinuosa* (Young & Duncan, 1994), *Trichophoromyia* sp. 1. of Araracuara (Morales & Minter, 1981), *Trichophoromyia velascoi* (Le Pont & Desjeux, 1992), *Trichophoromyia velezbernali* Posada-López, Galvis-Ovallos & Galati, 2018, *Trichophoromyia viannamartinsi* (Sherlock & Guitton, 1970) and *Trichophoromyia wilkersoni* (Young & Rogers, 1984).

*Etymology*: The name of the new subgenus *Dilermandomyia* subg. nov. is dedicated to our colleague José Dilermando de Andrade Filho for his great contribution to the taxonomy of New World sand flies.

*Reburramyia* gen. nov. Rodrigues & Galati (Fig. 3h–n)

LSID: urn:lsid:zoobank.org:act:58F23F61-325B-4979-94AF-AE2FAFBD8E72 

Type-species: *Phlebotomus reburrus* Fairchild & Hertig, 1961.

**Diagnosis**. **Both sexes**: all flagellomeres covered with many simple setae (> 10 per segment); absence of Newstead’s sensilla on palpus 2; lacinia of the maxilla with a single row of external teeth. **Female**: cercus long, length twofold or more its width; terminal knob of the spermathecae long, approximately threefold longer than its width; individual spermathecal ducts with subtle outgrowths.

Composition–one species: *Reburramyia reburra* (Fairchild & Hertig, 1961) gen. nov., comb. nov.

Comments: The holotype and paratypes of *Reburramyia reburra* gen. nov., comb. nov. originate from Panama. Its distribution extends from the west and north of the Andes Mountain range, while the vast majority of *Trichophoromyia* spp. occupy forest environments in the Amazon biome. One male and one female of the type series are deposited in the Museum of Zoology of the University of São Paulo, Brazil, and these were analyzed in this study to propose this new genus. Both specimens are in relatively good mounting condition, but the female spermathecae appear to be desiccated, which can happen during the sand fly clarification process. Because of this, the illustration of some characters, such as the length, excrescences of the duct and number of rings, was difficult. However, it was possible to confirm the morphological observations from the original illustrations by Fairchild and Hertig [57], and the illustrative dichotomous key by Young and Duncan [58].

*Etymology*: The name of the new genus *Reburramyia* gen. nov. is derived from the Latin name reburrus (one with bristling hair), referencing to the many bristling setae on the flagellum, the main diagnosis among other groups within the Psychodopygina subtribe.

**Fig. 4:**
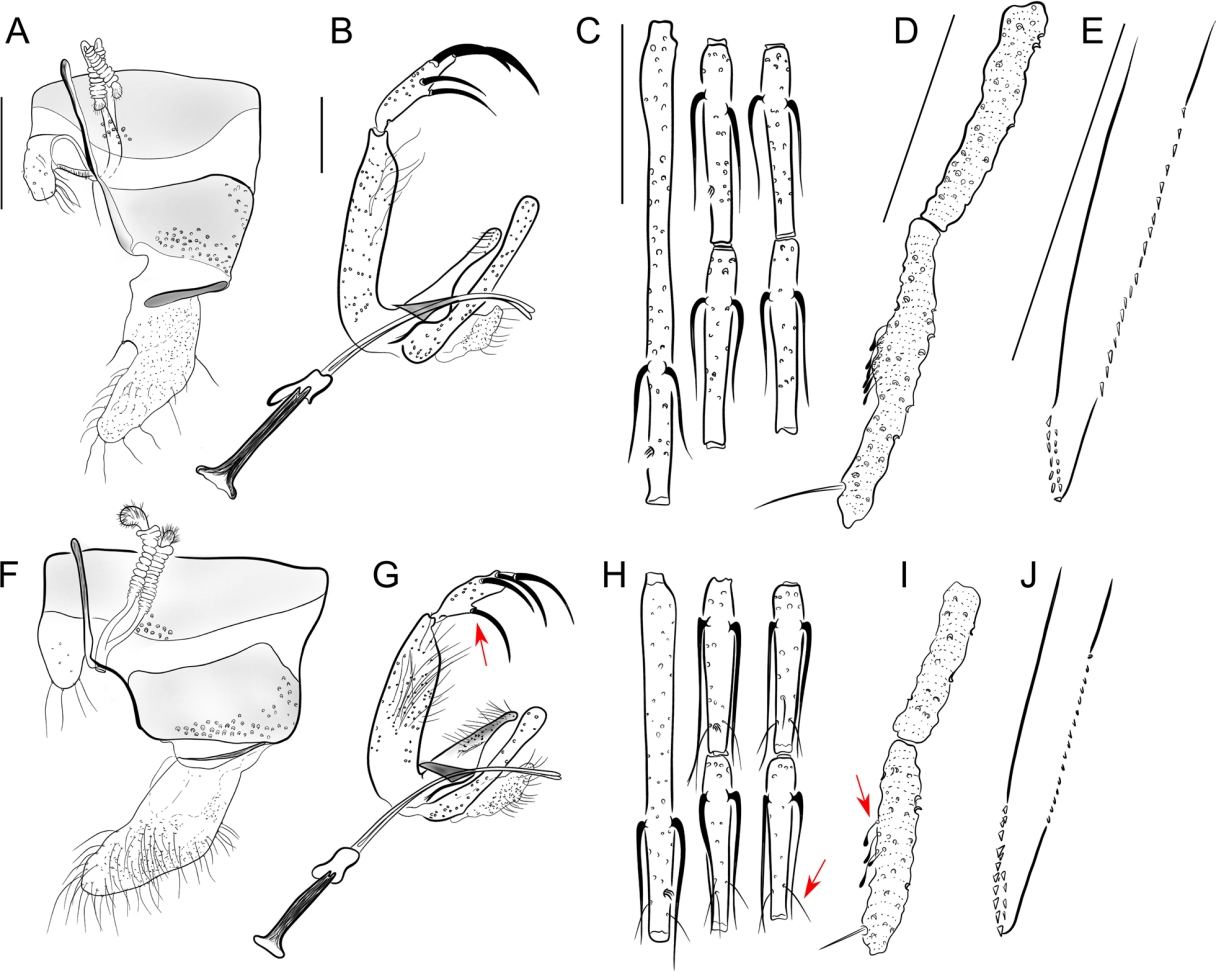
Main morphological characters used to identify sand flies of the genera *Nyssomyia* (**A**–**E**) and *Shawmyia* (**F–J)**. **A** and **F** Female spermathecae of *Ny. intermedia* and *Sh. richardwardi*, respectively. **B**, **G** Male terminalia. **C**, **H** Male flagellomeres FI to FV. **D** and **I** Male palpi P2 and P3, respectively, with Newstead’s sensilla. **E**, **J** Female lacinia of the maxilla. Red arrows indicate diagnostic characters for the identification of the genus *Shawmyia*. Different illustrations of the same structure are in the same scale. Scale bars: 100 μm. PP, Posterior probability

*Nyssomyia* Barretto, 1962 (Fig. 4a–e)

Type-species: *Phlebotomus intermedius* Lutz & Neiva, 1912.

**Diagnosis**. **Both sexes**: absence of preapical papilla in flagellomeres FIII; absence of Newstead’s sensilla in palpus 2; implantation of Newstead’s sensilla on palpus 3 grouped in a slightly swollen area near the middle of the segment; presence of few simple setae only in the apical flagellomeres FXII–FXIV; lacinia of the maxilla with two rows of external teeth. **Males**: terminalia shorter than the mesonotum length; gonostyle with long spines, the apical one equivalent to the length of the gonostyle; the internal spine of the gonostyle implanted in the middle or distal third of the segment. **Females**: cercus long, length twofold or more its width; spermathecae ringed, with approximately 5–15 rings, with the apical one equivalent to or slightly longer than the preapical one.

Composition–19 species: *Nyssomyia anduzei* (Rozeboom, 1942), *Nyssomyia antunesi* (Coutinho, 1939), *Nyssomyia bibinae* (Léger & Abonnenc, 1988), *Nyssomyia delsionatali* Galati & Galvis, 2012, *Nyssomyia edentula* (León, 1971), *Nyssomyia elongata* (Floch & Abonnenc, 1945), *Nyssomyia fraihai* (Martins, Falcão & Silva, 1979), *Nyssomyia hernandezi* (Ortiz, 1965), *Nyssomyia intermedia* (Lutz & Neiva, 1912), *Nyssomyia neivai* (Pinto, 1926), *Nyssomyia singularis* (Costa Lima, 1932), *Nyssomyia sylvicola* (Floch & Abonnenc, 1945), *Nyssomyia trapidoi* (Fairchild & Hertig, 1952), *Nyssomyia umbratilis* (Ward & Fraiha, 1977), *Nyssomyia urbinattii* Galati & Galvis, 2012, *Nyssomyia whitmani* (Antunes & Coutinho, 1939), *Nyssomyia ylephiletor* (Fairchild & Hertig, 1952), *Nyssomyia yuilli yuilli* (Young & Porter, 1972), *Nyssomyia yuilli pajoti* (Abonnenc, Léger & Fauran, 1979).

*Shawmyia* gen. nov. Rodrigues & Galati (Fig. 4f–j)

LSID: urn:lsid:zoobank.org:act:1C338BEC-573B-4810-A211-B0CE531A8AAD

Type-species: *Lutzomyia richardwardi* Ready & Fraiha, 1981.

**Diagnosis**. **Both sexes**: preapical papilla absent on flagellomeres FIII; Newstead’s sensilla absent on palpus 2; implantation of Newstead’s sensilla on palpus 3 scattered and without swollen area; presence of simple setae in all flagellomeres; lacinia of the maxilla with two rows of external teeth. **Males**: terminalia shorter than the mesonotum length; gonostyle with long spines, the apical one equivalent to the length of the gonostyle; internal spine of the gonostyle basally implanted and isolated. **Females**: cercus long, its length at least twofold its width; spermathecae ringed, with approximately 10–15 rings, with the apical one equivalent to or slightly longer than the preapical one.

Composition–two species: *Shawmyia richardwardi* (Ready & Fraiha, 1981) gen. nov., comb. nov., and *Shawmyia shawi* (Fraiha, Ward & Ready, 1981) gen. nov., comb. nov.

*Etymology*: The name of the new genus *Shawmyia* gen. nov. is dedicated to the Professor Jeffrey J. Shaw, for his esteemed contribution to the knowledge of sand flies and leishmaniasis.

**Table 2:** Identification key of genera and subgenera of the Psychodopygina subtribe

1	**♂/♀**: ascoids with evident posterior prolongation, or if they are rudimentary, their insertion in the article is pedunculated; first metatarsomere longer than or equivalent to the total sum of the other tarsomeres	*Psathyromyia* Barretto, 1962 .....2
	**♂/♀**: ascoids without developed posterior prolongation, if there is a prolongation it is rudimentary or not pedunculated; ratio of 1 st metatarsomere and the total length of the other ones variable	5
2	**♂/♀**: simple setae absent from flagellomere I and/or II and/or III **♂**: frequently, Newstead’s sensilla present on the second palpal segment **♀**: Newstead’s sensilla present on the second palpal segment	3
	**♂/♀**: generally, simple setae are present on flagellomere I and/or II and/or III; Newstead’s sensilla absent in second palpal segment	4
3	**♂/♀**: ascoids with long posterior prolongation, in flagellomere FII they reach its base **♂**: parameral sheath short, its length less than twice the width of its base; gonostyle with all the spines clearly implanted beyond its middle **♀**: spermathecae with distinct rings; common spermathecal ducts as long as 1.5-fold the individual duct	*Psathyromyia pifanoi* (Ortiz, 1972)
	**♂/♀**: ascoids with posterior prolongation at most reach the middle part of the region between their implantation and the base of flagellomere FII **♂**: parameral sheath long, its length equivalent to twofold or more the width of its base **♀**: vesiculous, tubular or ringed spermathecae, but in this latter case the rings are lightly demarcated, the individual ducts are very long and the common duct is rudimentary	*Psathyromyia *(*Forattiniella*) Vargas, 1978
4	**♂/♀**: papillae present on flagellomeres X–XI **♂**: gonocoxite with sclerotized setae located in its apical region **♀**: fifth palpal segment shorter than or equivalent to the third; spermathecae ringed, some of them being distinctively imbricated	*Psathyromyia (Xiphopsathyromyia)* Ibánez-Bernal & Marina 2015
	**♂/♀**: papillae absent from flagellomeres X–XI **♂**: gonocoxite without sclerotized setae in its apical region **♀**: 5th palpal segment longer than the third	*Psathyromyia (Psathyromyia) s. str*
5	**♂**: gonostyle with the upper external spine implanted in a conspicuous tubercle located in or before its middle and the lower external one implanted in a smaller tubercle situated more proximally or at the base of the former tubercle; paramere simple with differentiated setae in the apical region of its dorsal margin **♀**: spermathecae bulbous and enclosed in a sclerotized sheath	*Viannamyia* Mangabeira, 1941
	**♂**: gonostyle with the external upper spine implanted on its apical third and the external inner spine in or before its middle; paramere simple or lobed, with or without differentiated setae **♀**: spermathecae ringed	6
6	**♂/♀**: fifth palpal segment longer than the third; clypeus longer than or equivalent to two thirds of eye length	*Martinsmyia* Galati, 1995
	**♂**: clypeus short, equivalent to or smaller than one half the eye length **♀**: fifth palpal segment shorter than or equivalent to the third; clypeus of variable length	7
7	**♂/♀**: generally, presence of setae in the anterior region of katepisternum; mesonotum, generally, two-colored (posterior part of scutum and scutellum straw-colored, in contrast to the rest which is brown) **♂**: gonostyle with four well-developed spines, the inner one situated on its apical third **♀**: lacinia of the maxilla with a single row of external teeth; clypeus very long, equivalent to the length of the eye	*Bichromomyia* Galati, 1995
	**♂/♀**: thorax without setae in the anterior region of katepisternum; color of mesonotum variable **♂**: gonostyle with variable numbers of spines and position of the internal spine **♀**: generally, lacinia of the maxilla with two rows of external teeth; clypeus shorter than eye length	8
8	**♂/♀**: flagellomere I presenting two or more papillae **♂**: sum of fourth and fifth palpal segments smaller than or equivalent to the third **♀**: cibarium with two or more pairs of posterior teeth; spermathecae with all rings imbricated	*Psychodopygus* Mangabeira, 1941
	**♂/♀**: flagellomere I presenting only the pre-apical papilla **♂**: sum of the fourth and fifth palpal segments greater than the 3rd **♀**: cibarium with ≥ 3 pairs of posterior teeth; spermathecae rings with some or all non-imbricated	9
9	**♂/♀**: generally, presence of Newstead’s sensilla in the second palpal segment. **♀**: spermathecae with ≥ 25 rings; cercus short, length less than twofold its width	*Trichophoromyia* .....10
	**♂/♀**: Newstead’s sensilla absent in the second palpal segment **♀**: spermathecae with 5–20 rings, the apical ring equivalent to or slightly longer than the pre-apical one; cercus long, length twofold or more its width	11
10	**♂**: aedeagal ducts ≤ threefold the length of the sperm pump **♀**: spermathecae: apical ring ≤ threefold as long as the pre-apical ring	*Trichophoromyia (Trichophoromyia) s. str.*
	**♂**: aedeagal ducts > fourfold the length of the sperm pump **♀**: spermathecae: apical ring ≥ sixfold as long as the pre-apical ring	*Trichophoromyia* (*Dilermandomyia*) subg. nov.
11	**♂/♀**: presence of many simple setae (> 10) on all flagellomeres; lacinia of the maxilla with a single row of external teeth	*Reburramyia reburra* gen. nov., comb. nov.
	**♂/♀**: presence of few simple setae (2–3) on all flagellomeres or only on the apical ones; lacinia of the maxilla with 2 rows of external teeth	12
12	**♂/♀**: absence of simple setae on flagellomere I; Newstead’s sensilla grouped in a slightly swollen area near to the middle of P3 **♂**: implantation of the internal spine of the gonostyle in the middle or distal third of the segment	*Nyssomyia*
	**♂/♀**: presence of simple setae in flagellomere I; Newstead’s sensilla in P3 scattered, without swollen area **♂**: implantation of the internal spine of the gonostyle isolated in the basal region	*Shawmyia* gen. nov.

### Molecular taxonomy, and species delimitation—COI dataset

We generated 141 new *COI* sequences for 17 nominal species of *Trichophoromyia*, in addition to *Sh. richardwardi* gen. nov., comb. nov. (4 sequences) and a number of isomorphic *Trichophoromyia* females identified at genus level (7 sequences). Of these, we sequenced for the first time specimens of *Th. ruii*, *Th. readyi*, *Th. melloi*, *Th. iorlandobaratai*, *Th. gibba*, *Th. flochi*, *Th. eurypyga*, *Th. castanheirai* and *Th. adelsonsouzai*. After assembling the complete *COI* dataset (i.e. our 152 samples merged with previously available sequences from GenBank), we obtained a final alignment of 254 *COI* barcodes (Additional file 1: Table S1), with a minimum coverage of 601 bp.

For the *Trichophoromyia* sequences, the intraspecific and interspecific pairwise distances ranged from 0.0% to 5.02% and from 0.0% to 11.7%, respectively. *Trichophoromyia auraensis* has the largest value (5%), followed by *Th. ininni* (4.26%) and *Th. brachipyga* (4.1%) (Table 3). In comparison, the species pairs with the lowest interspecific distances (also sharing *COI* haplotypes) were *Th. auraensis*/*Th. velezbernali* and *Th. castanheirai*/*Th. peixotoi*. For *Nyssomyia*, the intraspecific and interspecific distances ranged from 0.0% to 2.28% and from 0.46% to 14.7%, respectively. The highest intraspecific distance was found for *Ny. whitmani* sequences, while the lowest distance to the nearest neighbor (i.e. the minimum interspecific distance) was found for the species pair *Ny. antunesi*/*Ny. urbinattii*.

**Table 3:** Sand fly species of the Psychodopygina subtribe used in this study for DNA barcoding analysis of cytochrome* c* oxidase subunit I gene

Species	*n* (This study/GenBank)^a^	Maximum (mean) intraspecific distance	Congeneric nearest neighbor	Nearest neighbor disance^b^
*Th. viannamartinsi* (Sherlock & Guitton, 1970)	20/5	2.58% (1.11%)	*Th. melloi*	3.65%
*Th. velezbernali* Posada-López, Galvis & Galati, 2018	–/3	0.67% (0.44%)	*Th. auraensis*	0.00%
*Th. ubiquitalis* (Mangabeira, 1942)	15/5	4.1% (2.06%)	*Th.* sp. of Amapá	5.71%
*Th.* sp. of Amapá (Cavalcante et al. *in prep*)	10/–	0.3% (0.06%)	*Th. ubiquitalis*	5.71%
*Th. ruii* (Arias & Young, 1982)	2/–	0.3% (0.30%)	*Th. loretonensis*	0.15%
*Th. readyi* (Ryan, 1986)	20/–	2.74% (1.33%)	*Th. flochi*	0.46%
*Th. peixotoi* Rodrigues, Pinto & Galati, 2023	2/4	1.44% (0.90%)	*Th. castanheirai*	0.00%
*Th. octavioi* (Vargas, 1949)	9/3	1.82% (0.63%)	*Th. auraensis*	1.06%
*Th. melloi* (Causey & Damasceno, 1945)	9/–	3.04% (1.72%)	*Th. readyi*	0.61%
*Th. loretonensis* (Llanos, 1964)	–/1	n/c	*Th. ruii*	0.15%
*Th. jariensis* Cavalcante, Rodrigues & Galati, 2024	–/5	1.06% (0.67%)	*Th. adelsonsouzai*	0.61%
*Th. iorlandobaratai* Vasconcelos dos Santos, Santos Neto, Sánchez Uzcátegui & Galardo, 2018	6/–	1.22% (0.82%)	*Th. brachipyga*	1.82%
*Th. ininii* (Floch & Abonnenc, 1943)	–/2	4.26% (4.26%)	*Th. flochi*	0.91%
*Th. howardi* (Young, 1979)	–/2	0.00% (0.00%)	*Th. clitella*	1.33%
*Th. gibba* (Young & Arias, 1994)	8/–	0.76% (0.37%)	*Th. brachipyga*	1.52%
*Th. flochi* (Abonnenc & Chassignet, 1948)	5/–	1.67% (1.19%)	*Th. readyi*	0.46%
*Th. eurypyga* (Martins, Falcão & Silva, 1963)	6/–	0.61% (0.25%)	*Th. peixotoi*	1.91%
*Th. clitella* (Young & Pérez, 1994)	1/2	1.37% (1.02%)	*Th. melloi*	0.91%
*Th. castanheirai* (Damasceno, Causey & Arouck, 1945)	7/–	0.00% (0.00%)	*Th. peixotoi*	0.00%
*Th. brachipyga* (Mangabeira, 1942)	12/–	4.1% (2.06%)	*Th. adelsonsouzai*	0.76%
*Th. auraensis* (Mangabeira, 1942)	3/9	5.02% (2.68%)	*Th. velezbernali*	0,00%
*Th. adelsonsouzai* Santos, Silva, Barata, Andrade & Galati, 2013	6/–	0.61% (0.39%)	*Th. jariensis*	0.61%
*Re. reburra* (Fairchild & Hertig, 1961)	–/3	0.15% (0.10%)	*Th. eurypyga*	11.7%
*Sh. shawi* (Fraiha, Ward & Ready, 1981)	–/4	0.47% (0.28%)	*Sh. richardwardi*	4.89%
*Sh. richardwardi* (Ready & Fraiha, 1981)	4/4	1.68% (0.95%)	*Sh. shawi*	4.89%
*Ny. yuilli yuilli* (Young & Porter, 1972)	–/5	0.76% (0.36%)	*Ny. umbratilis*	2.43%
*Ny. yuilli pajoti* (Abonnenc, Léger & Fauran 1979)	–/3	0.67% (0.44%)	*Ny. urbinattii*	1.5%
*Ny. ylephiletor* (Fairchild & Hertig, 1952)	–/1	n/c	*Ny. trapidoi*	9.15%
*Ny. whitmani* (Antunes & Coutinho, 1939)	–/5	2.28% (1.49%)	*Ny. intermedia*	2.43%
*Ny. urbinattii* Galati & Galvis, 2012	–/4	0.00% (0.00%)	*Ny. antunesi*	0.46%
*Ny. umbratilis* (Ward & Fraiha, 1977)	–/5	1.06% (0.70%)	*Ny. yuilli yuilli*	2.43%
*Ny. trapidoi* (Fairchild & Hertig, 1952)	–/5	2.13% (1.43%)	*Ny. ylephiletor*	9.15%
*Ny. neivai* (Pinto, 1926)	–/2	0.46% (0.46%)	*Ny. whitmani*	2.13%
*Ny. intermedia* (Lutz & Neiva, 1912)	–/5	1.06% (0.64%)	*Ny. whitmani*	2.43%
*Ny. fraihai* (Martins, Falcão & Silva, 1979)	–/5	1.00% (0.50%)	*Ny. yuilli yuilli*	2.83%
*Ny. delsionatali* Galati & Galvis, 2012	–/3	0.00% (0.00%)	*Ny. antunesi*	1.06%
*Ny. antunesi* (Coutinho, 1939)	–/4	0.3% (0.23%)	*Ny. urbinattii*	0.46%
*Ny. anduzei* (Rozeboom, 1942)	–/3	0.91% (0.61%)	*Ny. yuilli yuilli*	3.8%

*Ny. Nyssomyia, Re. Reburramyia, Sh. Shawmyia, Th. Trichophoromyia*

^a^Number of sequences for each species

^ b^Minimum interspecific nearest neighbor distance

Regarding the dataset of specimens identified to species level (247 *COI* sequences; Table 3), 85.8% (*n* = 212) could be unambiguously identified by both BM and BCM criteria. Excluding the singletons of *Th. loretonensis* and *Ny. ylephiletor*, the correct identifications comprise all sequences of *Nyssomyia*, *Reburramyia* gen. nov., *Shawmyia* gen. nov. and partially sequences of *Trichophoromyia*. Ambiguous (8.09%) and incorrect (6.07%) identifications—for which the closest match is not conspecific—occurred for specimens of *Th. velezbernali*, *Th. auraensis*, *Th. peixotoi*, *Th. castanheirai*, *Th. flochi*, *Th. ruii*, *Th. brachipyga* and *Th. melloi*.

**Fig. 5:**
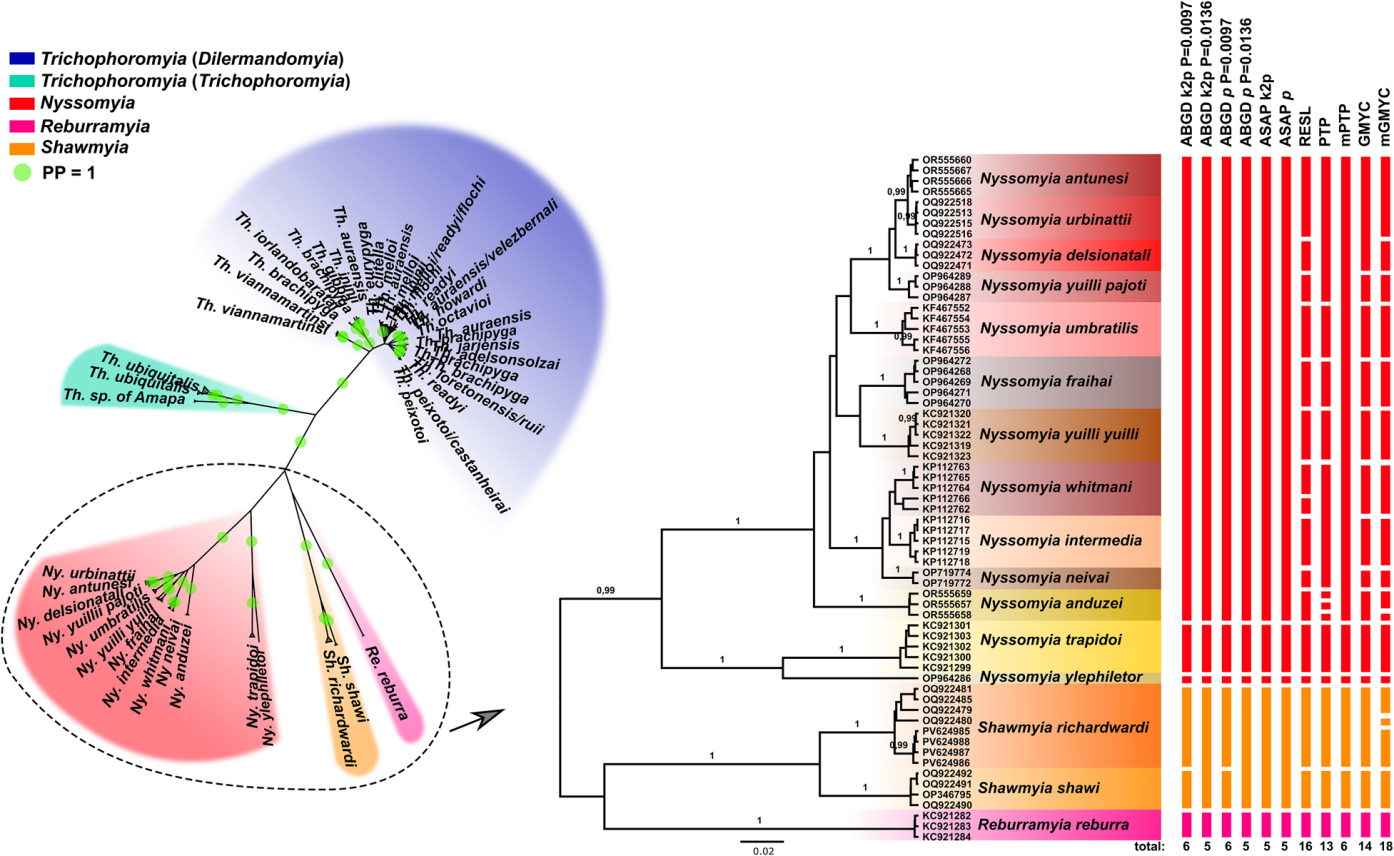
Phylogenetic gene tree (Bayesian Inference) based on cytochrome* c* oxidase subunit I gene (*COI*) sequences of *Trichophoromyia, Nyssomyia, Reburramyia,* and *Shawmyia*. Left: Unrooted tree highlighting the four main clades recovered. Right: Section of clades I and II showing the relationship of the sampled specimens. Terminal labels are GenBank accession numbers, and numbers near nodes are posterior probabilities > 0.95 (high support). Lateral bars indicate the different single-locus species delimitation scenarios and are colored according to the main clades of the unrooted tree. PP, Posterior probability

**Fig. 6:**
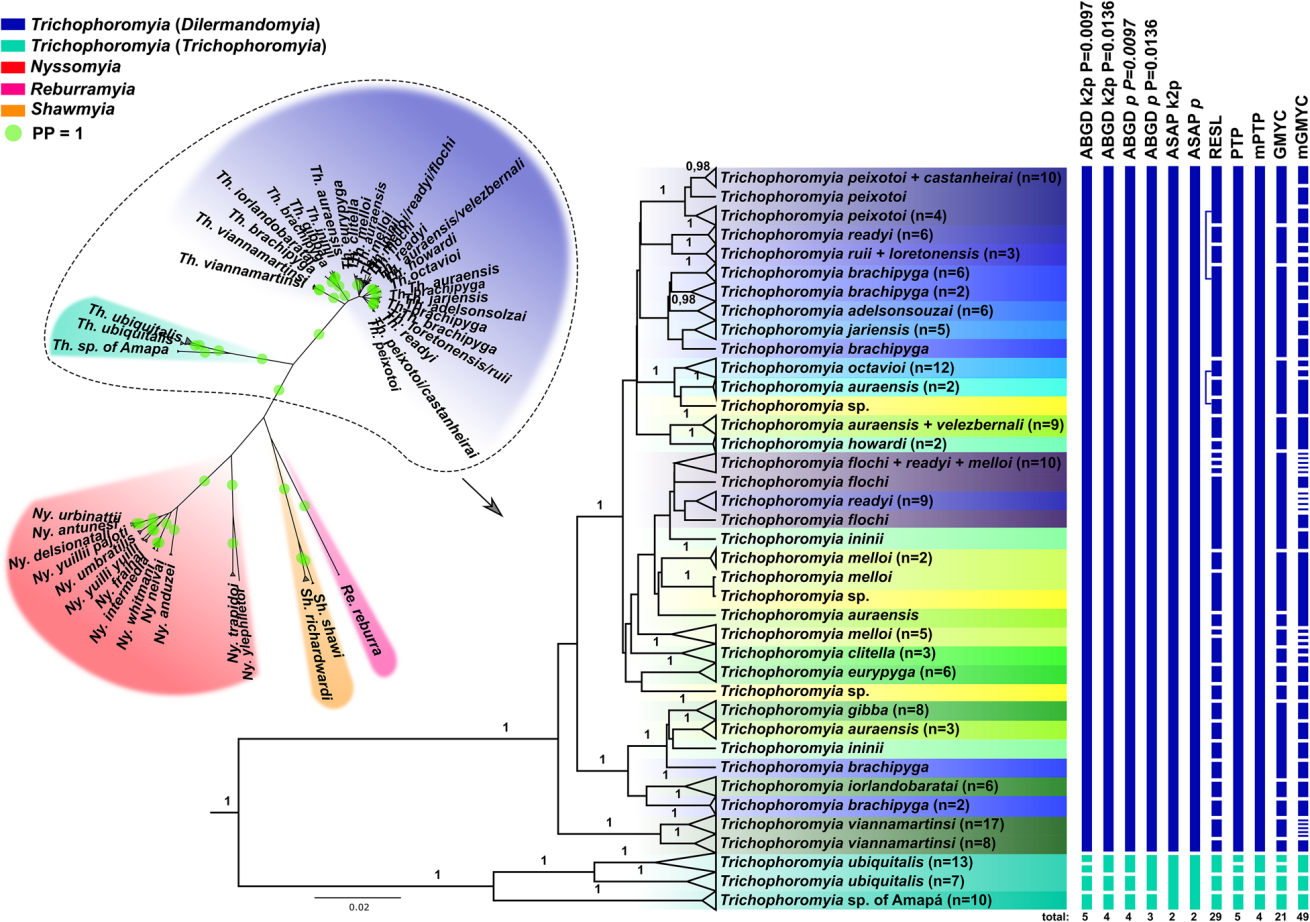
Phylogenetic gene tree (Bayesian Inference) based on cytochrome* c* oxidase subunit I gene (*COI*) sequences of *Trichophoromyia, Nyssomyia, Reburramyia,* and *Shawmyia*. Left: Unrooted tree highlighting the four main clades recovered. Right: Section of clades III (*Trichophoromyia* (*Trichophoromyia*)) and IV (*Trichophoromyia* (*Dilermandomyia*) subg. nov.) showing the relationship of the sampled specimens. Terminals are collapsed for some clades to improve visualization (full details in Additional file 1: Figure S1), and numbers near nodes are posterior probabilities > 0.95 (high support). Lateral bars indicate the different single-locus species delimitation scenarios and are colored according to the main clades of the unrooted tree

The phylogenetic gene tree of the *COI* dataset recovered four main clades: the first comprising species of the new proposed genera: *Re. reburra* gen. nov., comb. nov., *Sh. richardwardi* gen. nov., comb. nov. and *Sh. shawi* gen. nov., comb. nov. (Fig. 5); the second comprising all *Nyssomyia* spp. (Fig. 5); the third comprising *Th. ubiquitalis*, and *Th.* sp. of Amapá (i.e., *Trichophoromyia* (*Trichophoromyia*) *s. str.*) (Fig. 6); and the fourth comprising the remaining *Trichophoromyia* spp. of our dataset, which we propose here as a new subgenus named *Trichophoromyia* (*Dilermandomyia*) subg. nov. (Fig. 6). The first clade suggests that both species of *Shawmyia* gen. nov. are closely related, as evidenced by clustering in a well-supported subclade, and are also related to *Re. reburra* gen. nov., comb. nov. In fact, the positioning of these three species is ambiguous, as they appear to be related to the main *Trichophoromyia* clade regarding the maximum likelihood analysis, while they are related to the *Nyssomyia* clade in BI (despite not having high support values ​​for these basal groupings) (Additional file 1: Figure S1). In addition, the *COI* gene trees recovered well-supported clades for most of the species analyzed, mainly those of the taxa *Nyssomyia* and *Trichophoromyia* (*Trichophoromyia*). However, several specimens within *Trichophoromyia* (*Dilermandomyia*) subg. nov. clustered with non-conspecific individuals or reached a paraphyletic pattern in the tree. In this context, *Th. brachipyga*, *Th. readyi*, *Th. auraensis*, *Th. melloi* and *Th. ininii* stand out, being recovered in multiple regions of the tree, each being related (and merged into the same clade and/or MOTU) to different nominal species (Fig. 6; Additional file 1: Figure S1).

Single-locus species delimitation using discovery methods split the *COI* dataset into several MOTUs, but with large disagreement between methods. The number of MOTUs varied from seven to 67, with the most conservative methods being those based on pairwise distance matrices (ABGD and ASAP) and the coalescent tree-based PTP method, while RESL and GMYC had a greater tendency to partition sequences into many MOTUs (Figs. 5, 6). Most partitions merged several *Nyssomyia* species into the same MOTU, despite having reciprocal monophyletic clades (Fig. 5), and the less conservative methods seem to be more reliable in these cases. On the other hand, all species of *Trichophoromyia* (*Dilermandomyia*) subg. nov. (i.e. 20 different nominal species) were merged into the same MOTU with the most conservative algorithms, while RESL and GMYC split this clade into several MOTUs, but without concordance regarding nominal species, in most cases due to the lack of reciprocal monophyletic status of many of the species analyzed (Fig. 6).

### Species tree, time divergences, and validation—multilocus dataset

The amplification and sequencing of nDNA markers for *Trichophoromyia* spp. and *Sh. richardwardi* gen. nov., comb. nov. resulted in the analysis of 29, 24 and 28 sequences for *28S*, *ITS2* and *PARA*, respectively, which were merged with publicly available sequences from GenBank. For these markers (nDNA), we included some of the same species that had already been processed and analyzed for the *COI* dataset, in addition to *28S*-only sequences for *Th. velascoi*, *Th. pabloi*, *Th. cellulana* and *Th. sinuosa* processed by Zapata et al. [11].

**Fig. 7:**
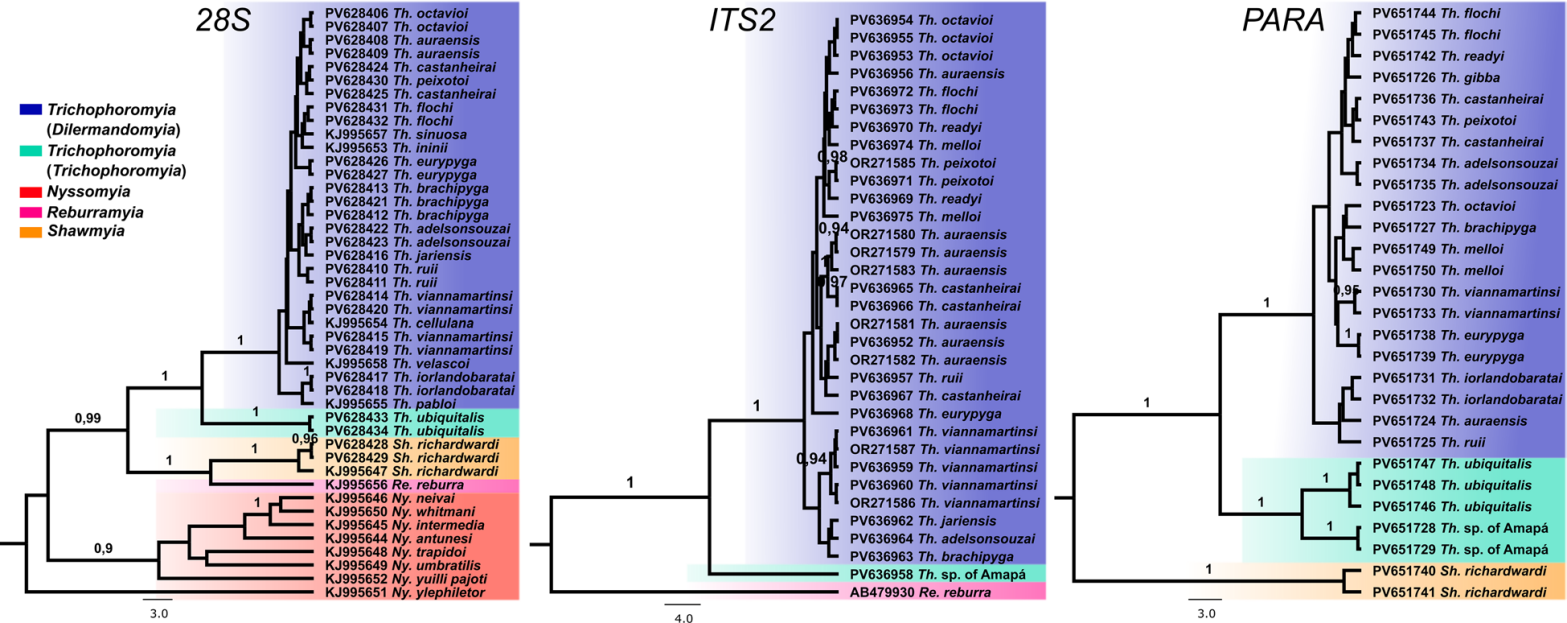
Phylogenetic gene trees (Bayesian Inference) based on nuclear DNA (nDNA) markers *28S*, *ITS2* and *PARA*. Terminal labels are GenBank accession numbers, and numbers near nodes are posterior probabilities > 0.90. *28S*, Large ribosomal subunit RNA gene; *ITS2*, internal transcribed spacer 2; *PARA*, paralytic gene

Phylogenetic gene trees were generated for each partition during species tree inference. The nDNA tree topologies showed a similar pattern to the *COI*-only dataset, despite large sample gaps regarding the species analyzed. All markers support the monophyly of *Trichophoromyia* (*Dilermandomyia*) subg. nov., in which several species also show paraphyletic patterns, although *Th. viannamartinsi*, *Th. peixotoi* and *Th. eurypyga* samples clustered in well-supported clades for the *ITS2* and/or *PARA* analysis (Fig. 7).

**Fig. 8:**
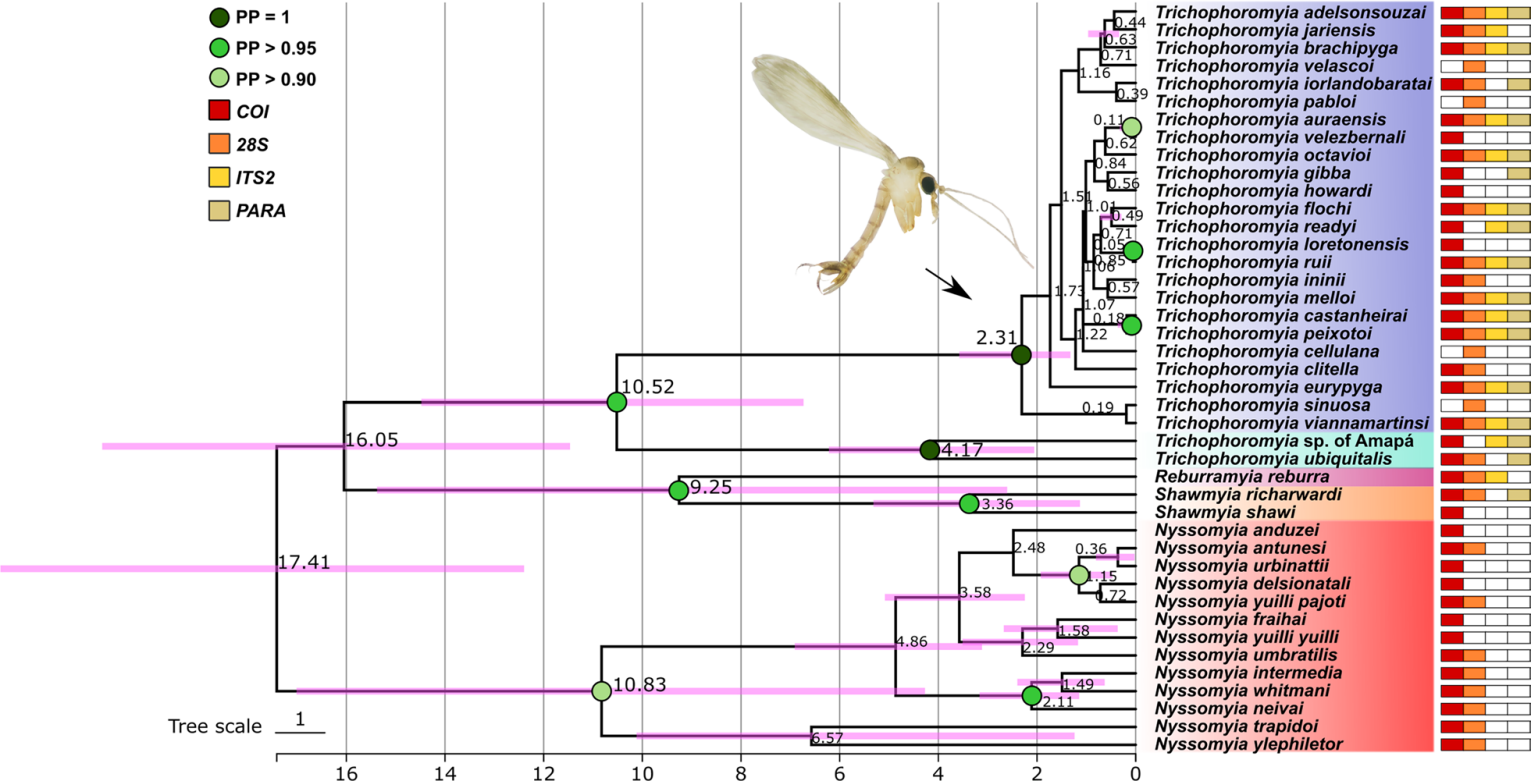
Time-calibrated species tree based on four molecular markers under the multispecies coalescent (MSC) model. Colored circles in nodes represent posterior probabilities. Numbers near nodes indicate mean divergence time in million years ago (mya). Pink node bars show divergence time estimation uncertainty. Vertical row of variously colored rectangles on the right refer to the molecular markers used for each taxon (shown in vertical row of similarly colored rectangles on the left). *COI* Cytochrome* c* oxidase subunit I gene, *28S*, large ribosomal subunit RNA gene; *ITS2*, internal transcribed spacer 2; *PARA*, paralytic gene; PP, posterior probability

The final alignments of nDNA markers in addition to our *COI* dataset were used to perform MSC species tree and species delimitation under a validation approach. The species tree based on the MSC model (Fig. 8) recovered some well-supported clades for our multilocus dataset that are in agreement with the *COI* gene tree inference, including the four major clades of *COI* analysis (Figs. 5, 6). However, the multilocus analysis shows high support values for the clade containing both *Shawmyia* gen. nov. species, and *Re. reburra* gen. nov. comb. nov., which are not well-supported to be related to either *Trichophoromyia* or *Nyssomyia*. *Trichophoromyia* has two main clades, referred to here as *Trichophoromyia* (*Trichophoromyia*) *s. str.*, and *Trichophoromyia* (*Dilermandomyia*) subg. nov., which differ in male and female morphological traits. For the former, only two species could be processed, but for the latter several species were sampled, which generally did not show well-supported groupings within it, except for the species pairs *Th. auraensis*/*Th. velezbernali*, *Th. loretonensis*/*Th. ruii* and *Th. castanheirai*/*Th. peixotoi* (Fig. 8).

The estimated divergence time indicates that the separation between the two *Trichophoromyia* subgenera may have occurred in the late Miocene at 10.52 mya (95% highest posterior density [HDP] interval: 6.73–14.48), and that multiple speciation events within *Trichophoromyia* (*Dilermandomyia*) subg. nov. began in the Early Pleistocene at 2.31 mya (95% HDP interval: 1.32–3.57) and have continued until more recently, with the last speciation events between *Th. castanheirai*/*Th. peixotoi* and *Th. ruii*/*Th. loretonensis* occurring around 180 kya and 50 kya, respectively (Fig. 8).

**Fig. 9:**
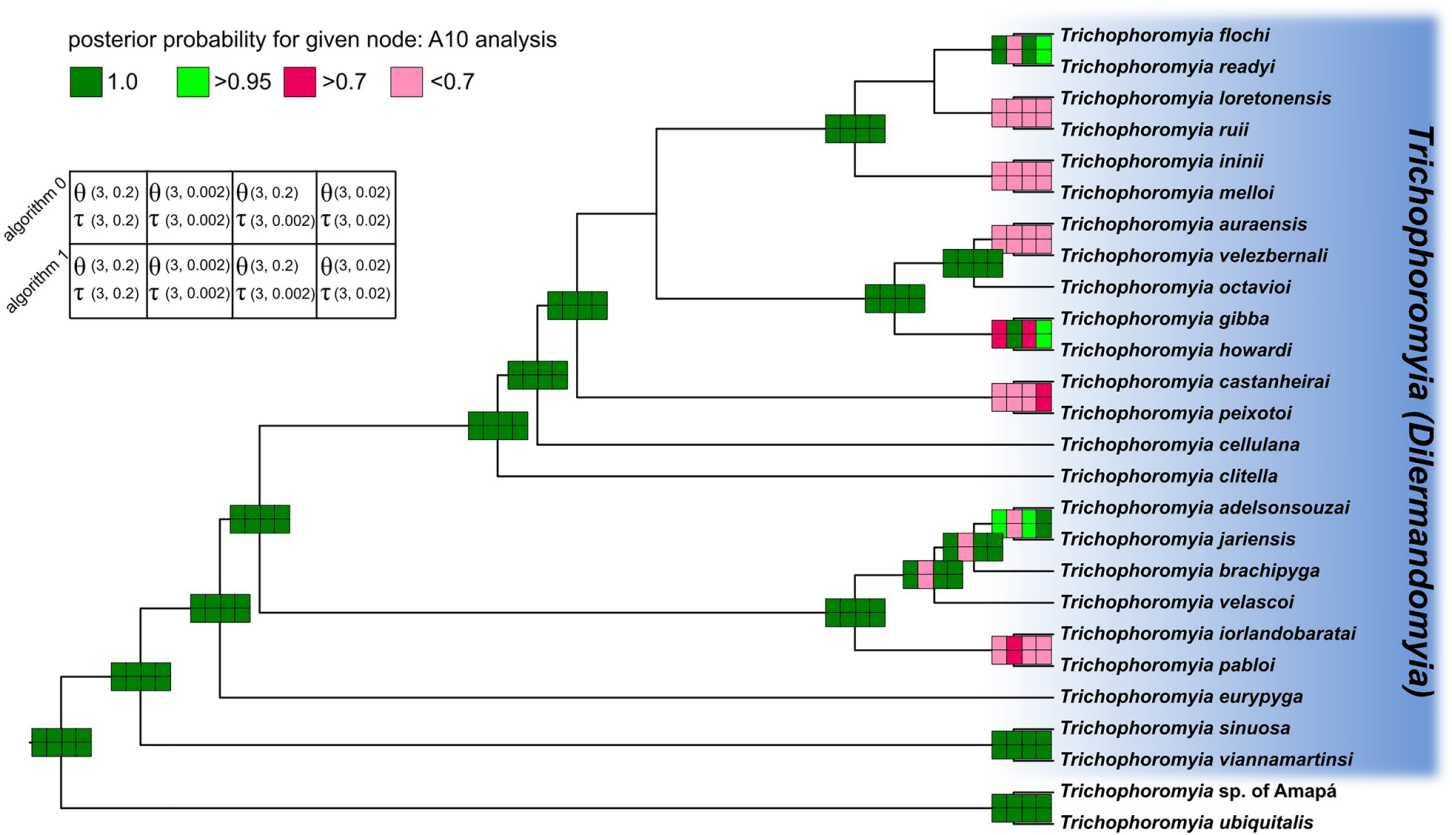
Species delimitation under the multispecies coalescent (MSC) model implemented in the Bayesian Phylogenetics and Phylogeography model (A10 analysis) for *Trichophoromyia*. The fixed guide tree topology follows StarBeast2 analysis. The boxes in nodes are color-coded according to the posterior probability (top left) of each node being a speciation event under different prior and algorithm schemes

Species delimitation under the MSC model and multilocus dataset showed that high posterior probabilities for some of the nodes of species tree may in fact be speciation events for *Trichophoromyia* (*Dilermandomyia*) subg. nov. (Fig. 9). There was large disagreement between delimitation scenarios across the different inverse-gamma (IG) prior settings, especially for θ values (population size), in which small ancestral population sizes (IG: 3, 0.002, with mean = 0.001) usually had low support values for speciation events of closely related taxa. We then considered the other prior schemes with larger ancestral population sizes as more reliable for our dataset. Regardless of the values ​​of θ and τ, the BPP did not consider the splits in these species pairs as valid (i.e. PP < 0.95): *Th. loretonensis*/*Th. ruii*, *Th. ininii*/*Th. melloi*, *Th. auraensis*/*Th. velezbernali*, *Th. castanheirai*/*Th. peixotoi* and *Th. iorlandobaratai*/*Th. pabloi*.

## Discussion

This study was the first to analyze molecular markers for a comprehensive number of species in the genus *Trichophoromyia*, which has been neglected in integrative taxonomy and systematics studies of sand flies [13]. In this study, we sought to provide phylogenetic and species delimitation inferences for 26 *Trichophoromyia* nominal species, which represent > 50% of the described taxa for this group, currently comprising 47 species (excluding *Re. reburra* gen. nov., comb. nov., from this list; or 48, considering *Th.* sp. of Amapá, as formerly described) [1, 2]. Consequently, even though our sampling effort signifies a substantial augmentation with respect to the most recent molecular analyses of the genus [14, 16], it is imperative to consider in our results the existing lacunae regarding the species and populations processed to obtain molecular markers. This highlights the need for more comprehensive and rigorous studies to better understand the complexity of diversity within *Trichophoromyia*, particularly in the context of other genera belonging to the Psychodopygina subtribe.

### Single-locus species identification and delimitation

A significant challenge in the study of sand flies arises from the presence of females that are morphologically indistinguishable. This is a relevant issue in sand fly taxonomy studies because females are hematophagous and thus act as biological vectors of pathogens, and the inability to distinguish specimens by taxon when more than one species with isomorphic females is present in a particular locality is a major limitation. Additionally, it can lead to ambiguous associations when natural infection of pathogens, such as *Leishmania* sp., is detected in sand flies [4, 59]. For example, all females within the sand fly genera *Pressatia* Mangabeira, 1942 and *Trichopygomyia* Barretto, 1962 are indistinguishable, and *Trichophoromyia* is not distant from this scenario, with most of its species being isomorphic despite the great diversity of males [7]. In our study, although many species are apparently indistinguishable based on their *COI* sequences, the association of females and males can be made for certain localities in order to provide appropriate morphological descriptions for the females. For instance, sampling efforts carried out in the Parque Nacional da Amazônia (PNA) allowed us to sample males of *Th. ubiquitalis*, *Th. readyi* and *Th. peixotoi*. It is assumed that the latter two are indistinguishable from each other [15], but our molecular analysis suggests distinct clades for both species, which would allow the association and description of females of both species in future studies.

The amplification and analysis of DNA barcoding fragments (*COI* gene) have been extensively evaluated for sand flies [13]. Typically, genetic divergences is smaller between individuals of the same species than between individuals of different species. This characteristic of *COI* sequences renders them a suitable marker for use as a barcode for species-level molecular identifications [18, 60, 61]. However, our sequence distance analyses for *Trichophoromyia* challenges this assumption, as more than half of the analyzed species have nearest-neighbor distances that are lower than the maximum intraspecific ones (Table 3). Indeed, the overlap of these classes of distances is to be expected for datasets composed of closely related species, given that the presence of the “barcoding gap” has been associated with studies exhibiting poor taxon sampling [62–65].

The admixture pattern of different classes of genetic distances also prevents species-level reciprocal monophyly regarding gene trees. This outcome, disregarding operational bias such as incorrect morphological assignment, may be the result of biological phenomena such as incomplete lineage sorting, introgression and hybridization [66, 67]. The occurrence of species-level paraphyly is actually not particularly uncommon, with approximately 20% of arthropod species exhibiting this pattern in phylogenetic gene trees [66, 68]. For sand flies, studies reporting a high efficiency of the *COI* barcodes in species delimitation and the reconstruction of monophyletic clades in gene trees may also be the result of poor sampling in terms of closely related species. In this study, we sought to sample close-related *Trichophoromyia* spp., and the results demonstrated high levels of paraphyly, similar to that demonstrated for the subgenus *Evandromyia* (*Aldamyia*) [10, 69].

High rates of para- and polyphyly in *COI* gene trees pose a significant challenge in the single-locus species delimitation process when discovery algorithms are used, as the reciprocal monophyly or the presence of a barcode gap is an essential criterion for accurately delineating MOTUs [70]. Our findings support this assumption, showing high rates of discordance of single-locus partition schemes and the morphological delimitation of the samples. Some algorithms overlump (ABGD, ASAP and PTP) or oversplit (RESL and GMYC) many species of our dataset (Fig. 6), showing clear patterns of MERGE (i.e. a species placed in a single MOTU together with individuals of another species, as stated by Ratnasingham and Hebert [40]), such as that found for *Th. castanheirai* and *Th. velezbernali*, and MIXTURE (a species assigned to more than one MOTU with at least one of them admixed with nonspecific taxa), as found for *Th. peixotoi*, *Th. auraensis*, *Th. readyi*, *Th. flochi* and *Th. melloi*. Discordances among algorithm partitions are somewhat expected; GYMC appears to oversplit *COI* datasets, identifying multiple MOTUs in gene trees even when there is little divergence between sequences [71], and the distance-based methods ABGD and ASAP may overlump morphologically distinct species when lineage divergence is too low [55, 70].

On the other hand, *COI* analysis also shows SPLIT patterns (a single nominal species comprising at least two different MOTUs), which are in accordance with geographically isolated populations. This is the case for *Th. auraensis* and *Th. viannamartinsi*. The former species formed three well-structured clades/MOTUs: one for specimens from the Eastern Amazon (Maranhão), one for Western (Acre) [16] and one which we detected in our study—a grouping from the state of Amazonas, in the central region of the biome. Regarding the latter species, *Th. viannamartinsi*, its genetic structure also seems to be related to geographically distinct populations, being the first clade/MOTU encompassing sequences from the state of Bahia, whereas the second one was for specimens from Alagoas state. In addition to the evident geographic distance of these populations, the structuring of *Th. viannamartinsi* can be associated with the different centers of endemism of the Atlantic Forest, which are related to the divergence of different evolutionary lineages for various organisms [72]. In this context, the first group occupies the “Coastal Bahia” and the second one occupies the “Pernambuco” endemism center. These divergent lineages may be a cryptic species complex or simply highly divergent and historically isolated populations, and these results should be further explored using morphometrics and other lines of evidence to validate these patterns (e.g. [73]), although our initial morphological analysis did not indicate diagnostic morphological characteristics that would justify the description of new taxa.

The efficacy of *COI* barcodes in identifying *Trichophoromyia* based on species delimitation algorithms is not straightforward, mainly due to the proximity of the species in our dataset. Therefore, we also implemented a sequence-similarity and tree-free approach (BM and BCM criteria) to estimate this, which generally recovers good success rates even in cases of great species-level paraphyly [64, 74]. In the present study, seven of the 21 analyzed *Trichophoromyia* species (discarding the singleton of *Th. loretonensis*) shows ambiguous or misidentification status: *Th. velezbernali*, *Th. auraensis*, *Th. peixotoi*, *Th. castanheirai*, *Th. flochi*, *Th. ruii*, *Th. brachipyga* and *Th. melloi*. Consequently, from a pragmatic perspective of the DNA barcoding tool for species identification without the need for morphological investigation, the *COI*-only analysis is inadequate for *Trichophoromyia*. However, although the use of *COI* sequencing for species-level identification appears to be unreliable and unjustifiable for some species of this group, its analysis in conjunction with sequences of close-related groups, such as *Nyssomyia*, *Shawmyia* gen. nov., and *Reburramyia* gen. nov., has enhanced our understanding of the diversification and evolution of these taxa, particularly when analyzed using a multilocus dataset.

### Multilocus analyses

In order to ascertain whether the high levels of paraphyly can be considered a special case of mito-nuclear discordance [75], an evaluation of nDNA markers for *Trichophoromyia* was also performed. This discordance may be explained by incomplete lineage sorting, sex-biased dispersal, asymmetrical introgression, natural selection and/or *Wolbachia*-mediated genetic sweeps [76], leading to incongruent species delimitation scenarios [77, 78]. The sampling of non-mitochondrial gene fragments was limited in the present study, but the results also demonstrated that some of the *Trichophoromyia* spp. exhibiting paraphyletic patterns for *COI* also lack reciprocal monophyly for *28S*, *ITS2* and *PARA* markers, albeit not to the same extent as observed for *COI*. For example, *ITS2* exhibited a well-supported clade for both *Th. castanheirai* and *Th. peixotoi*, which were previously indistinguishable based on *COI*, whereas the *PARA* gene tree merged these two species into the same cluster (Fig. 7).

Both tree topologies and species delimitation partitions using single-locus data may be misleading because gene trees are not species trees [79–81]. The MSC model is a theoretical framework that enables the presence of heterogeneity among gene trees. It was developed to address the challenges posed by incongruences between gene trees and species trees by incorporating stochastic fluctuations in the genealogical history of sequences across the genome as a natural evolutionary process [44, 45, 82]. In this context, we estimated the species tree phylogeny using sequences of *COI*, *28S*, *ITS2* and *PARA* for specimens identified as both *Trichophoromyia* and *Nyssomyia*. In the context of basal relationships, the resulting topology yielded four major clades that are partially concordant with *COI* gene trees, thereby confirming the paraphyly of both genera, which lead us to the proposals of new ones, named here as *Reburramyia* gen. nov., and *Shawmyia* gen nov., respectively. In a recent investigation of the molecular phylogeny of the Psychodopygina subtribe, Zapata et al. [11] demonstrated the tricky positioning of *Sh. richardwardi* gen. nov., comb. nov. (formerly known as a *Nyssomyia* species), which was found to be more closely related to *Trichophoromyia* than to *Nyssomyia*. We reinforce this, and added sequences from its morphologically closest species, *Sh. shawi* gen. nov., comb. nov. In addition, *Re. reburra* gen. nov., comb. nov. (formerly known as a *Trichophoromyia* species) also have an uncertain phylogenetic positioning, being grouped with the previously mentioned species (Fig. 8). Following a thorough analysis of the morphological characteristics, we determined that these three taxa exhibit traits that are somewhat intermediate, displaying characteristics that are well-established for both genera (Table 1), leading us to argue that none of these were adequately allocated to their respective groups, both due morphological and molecular inconsistencies, thus enabling the proposal of their respective new genera.

This is the first proposal of new names for genus groups for New World sand flies based on an integrative taxonomy approach. The proposal of *Reburramyia* gen. nov. and *Shawmyia* gen. nov. increases the number of Phlebotominae Neotropical genera to 25, and nine for the Psychodopygina subtribe. This latter group is mainly characterized by the absence of ventro-cervical sensilla and a fourth palpal segment perceptibly smaller than the second one (Galati et al. [7]), and comprises most of the species involved in the transmission of cutaneous leishmaniasis agents, including *Sh. shawi* gen. nov., comb. nov. [6]. The diagnosis of both *Nyssomyia* and *Trichophoromyia* genera relies on morphological characters of male terminalia and female spermathecae. These elements were used by Galati [10] for the inclusion of *Re. reburra* gen. nov., comb. nov. into the *Trichophoromyia* group, while *Sh. richardwardi* gen. nov., comb. nov. and *Sh. shawi* gen. nov., comb. nov. were considered to be as members of *Nyssomyia* genus. However, the discordant results of molecular phylogeny have permitted the identification of morphological characteristics that could justify the establishment of new groups to accommodate the taxa in question (Table 1). In contrast to all *Trichophoromyia* spp., the genus *Reburramyia* gen. nov. exhibits notable differences in its female specimens, characterized by a reduced number of spermathecae rings, an elongated terminal knob, the absence of Newstead sensilla on P2 and a single row of external teeth on the lacinia of the maxilla (Fig. 3). Also, *Shawmyia* gen. nov. is distinguished from the other *Nyssomyia* spp. by the presence of simple setae in all basal flagellomeres, the location of Newstead sensilla on P3, the absence of a swollen area on P3 and the basal position of the internal spine of the gonostyle (Fig. 4). Therefore, despite morphological differences not usually used in the delimitation of different genera, the congruence of morphological and molecular data reinforces the hypothesis presented in this study.

We also determined that there was sufficient evidence to propose a new subgenus within *Trichophoromyia*, namely *Trichophoromyia* (*Dilermandomyia*) subg. nov., based on the monophyletic status of all molecular analyses of this study (both genes and species tree inferences) and morphological characters. The main clade of *Trichophoromyia* has a clear pattern of splitting into two subclades. The first of these subclades comprises species in which males have aedeagal ducts that are fourfold or larger than the length of the sperm pump (usually sixfold) and the females have spermathecae’ apical ring more than sixfold longer than the pre-apical ones. The second subclade consists of *Th. ubiquitalis*, and *Th.* sp. of Amapá, which show relatively short aedeagal ducts (less than threefold the sperm pump) for males and short spermathecae’ apical ring for females. These two species, in addition to other taxa showing the same morphological pattern, can be considered as *Trichophoromyia* (*Trichophoromyia*), which also includes *Th. meirai, Th. omagua* and *Th. uniniensis*, which still need further validation by analyzing their relationship at least for *COI* gene. In contrast, we propose that *Trichophoromyia* (*Dilermandomyia*) subg. nov. comprises the remaining 43 nominal species of the genus, although the consistency of this grouping may need improvements considering the great biological complexity in it.

The divergence time estimates indicate that the closely related species of *Trichophoromyia* (*Dilermandomyia*) subg. nov. began to diversify around 2.31 mya (Fig. 8). This phenomenon coincides with the Pleistocene epoch (2.6 million years ago to 11,000 years ago), a period marked by multiple global climate shifts. Indeed, a substantial number of empirical investigations have demonstrated that a considerable proportion of the most recent speciation and adaptive radiation events of numerous taxa in the Amazon basin must have taken place during the Pleistocene epoch, including the sand fly [83]. However, a divergence of opinions exists on the underlying causal factors [84–86]. The prevailing historical explanation for this phenomenon is the Pleistocene refugium hypothesis (PRH) [87], which proposes that the origins of numerous extant Neotropical species occurred after the Neogene era. This hypothesis attributes this diversification to environmental fluctuations driven by recurring cycles of cooling and warming, which may have led to the fragmentation of the Amazon rainforest as a result of the expansion of drier grass savannas. This ecological transformation subsequently facilitated the allopatric diversification of species associated with forests.

These events may have impacted the current diversity of *Trichophoromyia* spp. (in which, apart from *Th. viannamartinsi*, were all collected in the Amazon region for this study), as most of them occur exclusively in forested areas, although some of them are now also adapted to urban regions [88]. However, the use of the PRH as the sole criterion for explaining the Amazonian biodiversity has been criticized, as recent evidence suggests that savannah and open grassland ecosystems were not widespread in the Amazon, although they may have occurred in a more localized manner [84, 89, 90]. The complexity of the Amazon and *Trichophoromyia* is likely the result of an intricate mixture of paleogeographic and biological evolutionary events, resulting in several barriers for ecologically suitable areas for these taxa, promoting speciation events [86, 91]. For example, the major Amazonian rivers are considered to be the main force acting in the divergence among multiple lineages of *Ny. umbratilis*, which also began to diversify during this period [92, 93]. Furthermore, research on Old World sand flies supports the hypothesis that diversification of closely related taxa and/or populations occurred during glacial and interglacial periods of the Pleistocene epoch [94–96]. A more thorough examination of *Trichophoromyia* that, incorporates species distribution patterns and a more extensive array of molecular data could facilitate the reconstruction of the phylogeography of the group.

With regard to the sister species relationships within *Trichophoromyia* (*Dilermandomyia*) subg. nov., species delimitation remains challenging even when employing multilocus validation methods under the MSC model (Fig. 9). The BPP analysis showed some disagreement regarding different prior schemes, with one with small ancestral population sizes (θ–IG [3, 0.002], mean = 0.001) being unreliable to our dataset, while the different BPP algorithms and divergence time prior (*τ*) did not influence the delimitation [48, 73]. This sensitivity is expected because small θ values reduce population-level variation and merge closely related lineages. Even for scenarios considered to be more reliable, the posterior probability of speciation events that occurred for certain species pairs was found to be minimal: *Th. loretonensis*/*Th. ruii*, *Th. ininii*/*Th. melloi*, *Th. auraensis*/*Th. velezbernali*, *Th. castanheirai*/*Th. peixotoi* and *Th. iorlandobaratai*/*Th. pabloi*. This may have happened due to major sampling gaps, in which some taxa had only one sequence for a single locus (e.g. *Th. loretonensis* and *Th. pabloi*). Disregarding these ones, the close-relatedness of some pairs is somewhat expected due to morphological similarities. *Trichophoromyia auraensis* and *Th. velezbernali* can be distinguished only by slightly differences in gonocoxite bristles and paramere aspects [97]. In contrast, *Th. ininii* and *Th. melloi* both resemble a robust paramere dorsal lobe, but are easily distinguishable by their shape, while *Th. castanheirai* and *Th. peixotoi* are also quite different in term of paramere shape, with the former presenting a great number of long bristles on the dorsal lobe of the paramere and the latter not (Fig. 2). The BPP analysis has been demonstrated to exhibit a high degree of efficacy in delimiting species, even in cases where the dataset contains a limited number of loci or singletons [98]. However, this does not seem to be sufficient due to the great complexity and recent diversification history of *Trichophoromyia* (*Dilermandomyia*) subg. nov., which presents high degrees of paraphyly across the genome, and may be associated with high rates of intense introgressive hybridization, due to female similarities and the sympatric distribution of many species.

## Conclusions

The sand fly genus *Trichophoromyia* exhibits a complex diversification history. The results of the phylogenetic analysis corroborated the paraphyletic status of *Nyssomyia* and *Trichophoromyia*. These observations, in addition to morphological traits, allowed us to propose two new genera and a new subgenus for the Psychodopygina subtribe—*Reburramyia* gen. nov., *Shawmyia* gen. nov. and *Trichophoromyia* (*Dilermandomyia*) subg. nov. This latter species group is of particular interest due to the prevalence of species-level paraphyletic patterns in its gene trees, in addition to its apparent recent diversification history. However, the use of multiple molecular markers failed to resolve the species delimitation of morphologically well-established nominal species, underscoring the fragility of our data in the face of the intricate taxonomic relationships within *Trichophoromyia*. The relationship of its species remains a challenge for future studies that should explore a wider range of molecular and morphological data.

## Supplementary Information

**Additional File 1: Figure S1.** Maximum likelihoodand Bayesian Inferencephylogenetic gene trees based on *COI* sequences of *Trichophoromyia* and *Nyssomyia*. Tip labels are GenBank Accession numbers and species names. Red tip labels indicate samples processed in this study, and black ones were extracted from GenBank. Values near nodes are SH-aLRT/UFBoot supports greater than 80/95%, and posterior probabilities greater than 0.95for ML and BI trees, respectively.

**Additional File 2: Table S1.** Full details of *Trichophoromyia* (*Th*.), *Nyssomyia* (*Ny*.), *Reburramyia* (*Re*.), and *Shawmyia* (*Sh*.) sand flies analyzed in this study and their amplified molecular markers (GenBank accession numbers).

**Additional File 3: Table S2.** List of sand fly species morphologically analyzed in this study. Number of specimens analyzed for each species, locality, and collection in which the specimen can be found.

## Data Availability

All sequences obtained from the study were deposited in the GenBank database under the accession numbers for the following molecular markers COI (PV624881–PV625032) 28S (PV628406–PV628434), ITS2 (PV636952–PV636975) and PARA (PV651723–PV651750).

## Abbreviations

ABGD: Automatic Barcode Gap Discovery
ASAP: Assemble Species by Automatic Partitioning
BI: Bayesian inference
BPP: Bayesian Phylogenetics and Phylogeography
*COI*: Cytochrome* c* oxidase subunit I
GMYC: Generalized Mixed Yule-Coalescent
*ITS2*: Internal transcribed spacer 2
ML: Maximum likelihood
MOTU: Molecular operational taxonomic unit
*PARA*: Paralytic
PTP: Poisson Tree Processes
RESL: Refined single linkage
