# Correction: On the integrative taxonomy of *Trichophoromyia* Barretto, 1962 and its relationship with *Nyssomyia* Barretto, 1962 (Diptera, Psychodidae, Phlebotominae): species delimitation, phylogeny, genus and subgenus description

**DOI:** 10.1186/s13071-026-07316-1

**Published:** 2026-03-19

**Authors:** Bruno Leite Rodrigues, Thiago Vasconcelos dos Santos, Andreia Fernandes Brilhante, Israel de Souza Pinto, Elisene Gonçala Rocha, Antonio Marques Pereira Júnior, Glaucilene da Silva Costa, Kamila Pereira de França, Keison de Souza Cavalcante, Paloma Helena Fernandes Shimabukuro, Jansen Fernandes Medeiros, Eunice Aparecida Bianchi Galati

**Affiliations:** 1 Faculdade de Saúde Pública (FSP), Universidade de São Paulo (USP), São Paulo, SP Brazil; 2 Seção de Parasitologia, Instituto Evandro Chagas (IEC), Ananindeua, PA Brazil; 3 Centro de Ciências da Saúde E Do Desporto, Universidade Federal Do Acre (UFAC), Rio Branco, AC Brazil; 4 Instituto Federal de Educação, Ciência e Tecnologia Do Espírito Santo (IFES), Ibatiba, ES Brazil; 5 Secretaria de Estado de Educação Do Pará (SEDUC), Novo Progresso, PA Brazil; 6 Laboratório de Análise E Visualização de Dados de Saúde Pública,, Fiocruz Rondônia, Porto Velho, RO Brazil; 7 Laboratório Central de Saúde Pública de Rondônia (LACEN–RO), Porto Velho, RO Brazil; 8 Laboratório de Entomologia, Fiocruz Rondônia, Porto Velho, RO Brasil; 9 Vice-Presidência de Pesquisa E Coleções Biológicas, Fundação Oswaldo Cruz, Biobanco da Biodiversidade E Saúde da Fiocruz, Rio de Janeiro, RJ Brazil

**Correction: Parasites & Vectors (2026) 19:44** 10.1186/s13071-025-07155-6

Following publication of the article [1], the following errors were brought to the attention of the journal: the caption of Table 2 was a duplicate of the caption of Table 1, references 15 and 16 were swapped, the scientific name ‘Reburrus reburrus’ in the article needed to be replaced (‘Reburrus’ was already in use in a group of insects published by Daniels, G. (1987) [10.1071/it9870473]). The article has since been corrected (‘Reburrus reburrus’ has been replace by ‘Reburramyia reburra’, which has been registered by the authors as appropriate). The authors and the journal thank you for reading this erratum.
