# Supplementary figures and images for: On the integrative taxonomy of Trichophoromyia Barretto, 1962 and its relationship with Nyssomyia Barretto, 1962 (Diptera, Psychodidae, Phlebotominae): species delimitation, phylogeny, genus and subgenus description
Source: Parasit Vectors. 2025 Dec 19;19:44. doi: 10.1186/s13071-025-07155-6 (PMC12831354; doi:10.1186/s13071-025-07155-6)

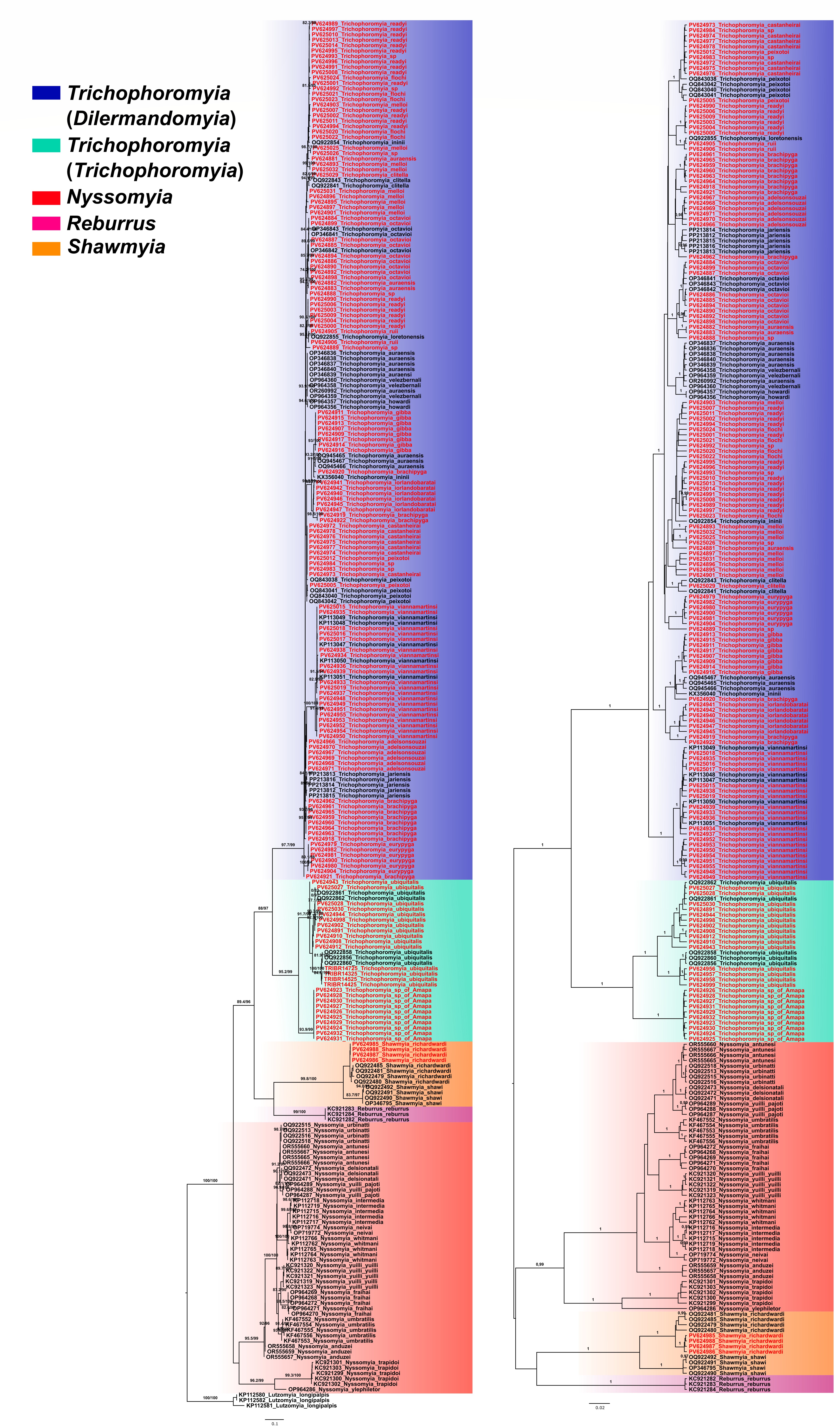

Supplement: Supplementary file 1 — Additional File 1: Figure S1. Maximum likelihoodand Bayesian Inferencephylogenetic gene trees based on COI sequences of Trichophoromyia and Nyssomyia. Tip labels are GenBank Accession numbers and species names. Red tip labels indicate samples processed in this study, and black ones were extracted from GenBank. Values near nodes are SH-aLRT/UFBoot supports greater than 80/95%, and posterior probabilities greater than 0.95for ML and BI trees, respectively. [file 13071_2025_7155_MOESM1_ESM.jpg]
