# Supplementary material for: On the integrative taxonomy of Trichophoromyia Barretto, 1962 and its relationship with Nyssomyia Barretto, 1962 (Diptera, Psychodidae, Phlebotominae): species delimitation, phylogeny, genus and subgenus description
Source: Parasit Vectors. 2025 Dec 19;19:44. doi: 10.1186/s13071-025-07155-6 (PMC12831354; doi:10.1186/s13071-025-07155-6)
Supplement: Supplementary file 2 — Additional File 2: Table S1. Full details of Trichophoromyia (Th.), Nyssomyia (Ny.), Reburramyia (Re.), and Shawmyia (Sh.) sand flies analyzed in this study and their amplified molecular markers (GenBank accession numbers). [file 13071_2025_7155_MOESM2_ESM.docx]

**Table S1.** Full details of Trichophoromyia (Th.), Nyssomyia (Ny.), Reburrus (Re.), and Shawmyia (Sh.) sand flies analyzed in this study and their amplified molecular markers (GenBank accession numbers).

| **Species** | **Sample ID** | **Museum ID** | **Country** | **State** | **Municipality** | **Lat** | **Long** | **Sex** | **Col. Date** | ***COI*** | ***28S*** | ***ITS2*** | ***PARA*** | **Reference** |
| --- | --- | --- | --- | --- | --- | --- | --- | --- | --- | --- | --- | --- | --- | --- |
| *Th. adelsonsouzai* | PA24 | 92254 | Brazil | Pará | Vitoria do Xingu | -3.416633 | -51.892750 | M | 24-May-2023 | PV624966 | PV628422 |  |  | This study |
| *Th. adelsonsouzai* | PA40 | 92255 | Brazil | Pará | Vitoria do Xingu | -3.416633 | -51.892750 | M | 24-May-2023 | PV624967 | PV628423 |  | PV651734 | This study |
| *Th. adelsonsouzai* | PA41 | 92256 | Brazil | Pará | Vitoria do Xingu | -3.416633 | -51.892750 | M | 24-May-2023 | PV624968 |  | PV636964 | PV651735 | This study |
| *Th. adelsonsouzai* | PA43 | 92257 | Brazil | Pará | Vitoria do Xingu | -3.416633 | -51.892750 | M | 24-May-2023 | PV624969 |  |  |  | This study |
| *Th. adelsonsouzai* | PA44 | 92258 | Brazil | Pará | Vitoria do Xingu | -3.416633 | -51.892750 | M | 24-May-2023 | PV624970 |  |  |  | This study |
| *Th. adelsonsouzai* | PA45 | 92259 | Brazil | Pará | Vitoria do Xingu | -3.416633 | -51.892750 | M | 24-May-2023 | PV624971 |  |  |  | This study |
| *Th. auraensis* | 4R42M11 | 91252b | Brazil | Acre | Brasiléia | -10.945556 | -68.703889 | M | n/a | OP346840 |  |  |  | Pinto et al. 2023 |
| *Th. auraensis* | 4R42M2 | 91244a | Brazil | Acre | Brasiléia | -10.945556 | -68.703889 | M | n/a | OP346836 |  |  |  | Pinto et al. 2023 |
| *Th. auraensis* | 4R42M24 | 91251c | Brazil | Acre | Brasiléia | -10.945556 | -68.703889 | M | n/a | OP346838 |  |  |  | Pinto et al. 2023 |
| *Th. auraensis* | 4R42M29 | 91263 | Brazil | Acre | Brasiléia | -10.945556 | -68.703889 | M | n/a | OP346837 |  |  |  | Pinto et al. 2023 |
| *Th. auraensis* | 4R42M7 | 91253a | Brazil | Acre | Brasiléia | -10.945556 | -68.703889 | M | n/a | OP346839 |  |  |  | Pinto et al. 2023 |
| *Th. auraensis* | AC303 | 92260 | Brazil | Acre | Xapuri | -10.443694 | -68.602167 | M | 22-Apr-2021 |  |  | OR271583 |  | Rodrigues et al. 2023 |
| *Th. auraensis* | AC307 | 92261 | Brazil | Acre | Xapuri | -10.443694 | -68.602167 | M | 22-Apr-2021 |  |  | OR271582 |  | Rodrigues et al. 2023 |
| *Th. auraensis* | AC309 | 92262 | Brazil | Acre | Xapuri | -10.443694 | -68.602167 | M | 22-Apr-2021 | OR260992 |  | OR271581 |  | Rodrigues et al. 2023 |
| *Th. auraensis* | AC310 | 92263 | Brazil | Acre | Xapuri | -10.443694 | -68.602167 | M | 22-Apr-2021 |  |  | PV636952 |  | This study |
| *Th. auraensis* | AC321 | 92264 | Brazil | Acre | Rio Branco | -10.072602 | -67.627792 | M | 20-Mar-2024 | PV624881 |  |  |  | This study |
| *Th. auraensis* | AM02 | 92265 | Brazil | Amazonas | Manaus | -3.086761 | -59.965853 | M | Jul-2014 | PV624882 | PV628408 | PV636956 |  | This study |
| *Th. auraensis* | AM04 | 92266 | Brazil | Amazonas | Manaus | -3.086761 | -59.965853 | M | Jul-2014 | PV624883 | PV628409 |  | PV651724 | This study |
| *Th. auraensis* | CAR360 | 92267 | Brazil | Maranhão | Governador Newton Bello | -3.445417 | -46.255556 | M | 18-Jan-2023 | OQ945467 |  | OR271580 |  | Rodrigues & Galati 2024 |
| *Th. auraensis* | CAR365 | 92268 | Brazil | Maranhão | Governador Newton Bello | -3.445417 | -46.255556 | M | 18-Jan-2023 | OQ945466 |  |  |  | Rodrigues & Galati 2024 |
| *Th. auraensis* | CAR370 | 92269 | Brazil | Maranhão | Governador Newton Bello | -3.445417 | -46.255556 | M | 18-Jan-2023 | OQ945465 |  | OR271579 |  | Rodrigues & Galati 2024 |
| *Th. brachipyga* | APB17 | 92270 | Brazil | Amapá | Pedra Branca do Amapari | 1.182892 | -52.890494 | M | 14-Jan-2014 | PV624918 | PV628412 |  | PV651727 | This study |
| *Th. brachipyga* | APB18 | 92271 | Brazil | Amapá | Pedra Branca do Amapari | 1.182892 | -52.890494 | M | 14-Jan-2014 | PV624919 | PV628413 |  |  | This study |
| *Th. brachipyga* | APB19 | 92272 | Brazil | Amapá | Pedra Branca do Amapari | 1.182892 | -52.890494 | M | 14-Jan-2014 | PV624920 |  |  |  | This study |
| *Th. brachipyga* | APB20 | 92273 | Brazil | Amapá | Pedra Branca do Amapari | 1.182892 | -52.890494 | M | 14-Jan-2014 | PV624921 |  |  |  | This study |
| *Th. brachipyga* | APB21 | 92274 | Brazil | Amapá | Pedra Branca do Amapari | 1.182892 | -52.890494 | M | 14-Jan-2014 | PV624922 |  |  |  | This study |
| *Th. brachipyga* | PA14 | 92275 | Brazil | Pará | Belém | -1.430208 | -48.456568 | M | 13-Mar-2023 | PV624959 |  |  |  | This study |
| *Th. brachipyga* | PA15 | 92276 | Brazil | Pará | Belém | -1.430208 | -48.456568 | M | 13-Mar-2023 | PV624960 | PV628421 | PV636963 |  | This study |
| *Th. brachipyga* | PA16 | 92277 | Brazil | Pará | Belém | -1.430208 | -48.456568 | M | 13-Mar-2023 | PV624961 |  |  |  | This study |
| *Th. brachipyga* | PA17 | 92278 | Brazil | Pará | Belém | -1.430208 | -48.456568 | M | 13-Mar-2023 | PV624962 |  |  |  | This study |
| *Th. brachipyga* | PA18 | 92279 | Brazil | Pará | Belém | -1.430208 | -48.456568 | M | 13-Mar-2023 | PV624963 |  |  |  | This study |
| *Th. brachipyga* | PA19 | 92280 | Brazil | Pará | Belém | -1.430208 | -48.456568 | M | 13-Mar-2023 | PV624964 |  |  |  | This study |
| *Th. brachipyga* | PA20 | 92281 | Brazil | Pará | Belém | -1.430208 | -48.456568 | M | 13-Mar-2023 | PV624965 |  |  |  | This study |
| *Th. castanheirai* | PAB01 | 92282 | Brazil | Pará | Belterra | -2.880978 | -54.932743 | M | 28-Jan-2019 | PV624972 |  | PV636965 | PV651736 | This study |
| *Th. castanheirai* | PAB02 | 92283 | Brazil | Pará | Belterra | -2.880978 | -54.932743 | M | 28-Jan-2019 | PV624973 | PV628424 |  | PV651737 | This study |
| *Th. castanheirai* | PAB03 | 92284 | Brazil | Pará | Belterra | -2.880978 | -54.932743 | M | 28-Jan-2019 | PV624974 | PV628425 | PV636966 |  | This study |
| *Th. castanheirai* | PAB04 | 92285 | Brazil | Pará | Belterra | -2.880978 | -54.932743 | M | 28-Jan-2019 | PV624975 |  | PV636967 |  | This study |
| *Th. castanheirai* | PAB05 | 92286 | Brazil | Pará | Belterra | -2.880978 | -54.932743 | M | 28-Jan-2019 | PV624976 |  |  |  | This study |
| *Th. castanheirai* | PAB06 | 92287 | Brazil | Pará | Belterra | -2.880978 | -54.932743 | M | 28-Jan-2019 | PV624977 |  |  |  | This study |
| *Th. castanheirai* | PAB07 | 92288 | Brazil | Pará | Belterra | -2.880978 | -54.932743 | M | 28-Jan-2019 | PV624978 |  |  |  | This study |
| *Th. cellulana* | n/a | n/a | Ecuador | n/a | n/a | n/a | n/a | M | n/a |  | KJ995654 |  |  | Zapata et al. 2023 |
| *Th. clitella* | 03ERO | n/a | Brazil | Rondônia | Pimenta Bueno | n/a | n/a | M | n/a | OQ922841 |  |  |  | Costa et al. unpublished |
| *Th. clitella* | 69MRO | n/a | Brazil | Rondônia | Machadinho d`Oeste | n/a | n/a | M | n/a | OQ922843 |  |  |  | Costa et al. unpublished |
| *Th. clitella* | ROB52 | 92289 | Brazil | Rondônia | Candeias do Jamari | -8.954694 | -63.173316 | M | 15-Dec-2023 | PV625029 |  |  |  | This study |
| *Th. eurypyga* | AM01 | 92290 | Brazil | Amazonas | Manaus | -3.086761 | -59.965853 | M | Jul-2014 | PV624900 |  |  |  | This study |
| *Th. eurypyga* | AM08 | 92291 | Brazil | Amazonas | Labrea | -7.664059 | -65.069582 | M | Feb-2014 | PV624904 |  |  |  | This study |
| *Th. eurypyga* | PAB09 | 92292 | Brazil | Pará | Belterra | -2.880978 | -54.932743 | M | 28-Jan-2019 | PV624979 | PV628426 |  |  | This study |
| *Th. eurypyga* | PAB10 | 92293 | Brazil | Pará | Belterra | -2.880978 | -54.932743 | M | 28-Jan-2019 | PV624980 | PV628427 | PV636968 |  | This study |
| *Th. eurypyga* | PAB11 | 92294 | Brazil | Pará | Belterra | -2.880978 | -54.932743 | M | 28-Jan-2019 | PV624981 |  |  | PV651738 | This study |
| *Th. eurypyga* | PAB12 | 92295 | Brazil | Pará | Belterra | -2.880978 | -54.932743 | M | 28-Jan-2019 | PV624982 |  |  | PV651739 | This study |
| *Th. flochi* | ROB39 | 92296 | Brazil | Amazonas | Canutama | -7.874846 | -63.872774 | M | 03-Nov-2021 | PV625020 | PV628431 | PV636972 | PV651744 | This study |
| *Th. flochi* | ROB40 | 92297 | Brazil | Amazonas | Canutama | -7.874846 | -63.872774 | M | 03-Nov-2021 | PV625021 | PV628432 | PV636973 | PV651745 | This study |
| *Th. flochi* | ROB41 | 92298 | Brazil | Amazonas | Canutama | -7.874846 | -63.872774 | M | 03-Nov-2021 | PV625023 |  |  |  | This study |
| *Th. flochi* | ROB43 | 92299 | Brazil | Amazonas | Canutama | -7.874846 | -63.872774 | M | 03-Nov-2021 | PV625022 |  |  |  | This study |
| *Th. flochi* | ROB44 | 92300 | Brazil | Amazonas | Canutama | -7.874846 | -63.872774 | M | 01-Oct-2023 | PV625024 |  |  |  | This study |
| *Th. gibba* | APB01 | 92301 | Brazil | Amapá | Calcoene | 2.318833 | -51.653402 | M | 30-Nov-2021 | PV624907 |  |  |  | This study |
| *Th. gibba* | APB04 | 92302 | Brazil | Amapá | Calcoene | 2.318833 | -51.653402 | M | 30-Nov-2021 | PV624909 |  |  |  | This study |
| *Th. gibba* | APB06 | 92303 | Brazil | Amapá | Calcoene | 2.318833 | -51.653402 | M | 30-Nov-2021 | PV624911 |  |  | PV651726 | This study |
| *Th. gibba* | APB08 | 92304 | Brazil | Amapá | Calcoene | 2.318833 | -51.653402 | M | 30-Nov-2021 | PV624913 |  |  |  | This study |
| *Th. gibba* | APB09 | 92305 | Brazil | Amapá | Calcoene | 2.318833 | -51.653402 | M | 30-Nov-2021 | PV624914 |  |  |  | This study |
| *Th. gibba* | APB10 | 92306 | Brazil | Amapá | Calcoene | 2.318833 | -51.653402 | M | 30-Nov-2021 | PV624915 |  |  |  | This study |
| *Th. gibba* | APB11 | 92307 | Brazil | Amapá | Calcoene | 2.318833 | -51.653402 | M | 30-Nov-2021 | PV624916 |  |  |  | This study |
| *Th. gibba* | APB12 | 92308 | Brazil | Amapá | Calcoene | 2.318833 | -51.653402 | M | 30-Nov-2021 | PV624917 |  |  |  | This study |
| *Th. howardi* | AMA75.1 | n/a | Colombia | Amazonas | Leticia | -4.208056 | -69.943056 | M | n/a | OP964356 |  |  |  | Posada-López et al. 2023 |
| *Th. howardi* | AMCK11-15.3 | n/a | Colombia | Amazonas | Leticia | -4.208056 | -69.943056 | M | n/a | OP964357 |  |  |  | Posada-López et al. 2023 |
| *Th. ininii* | n/a | n/a | French Guiana | n/a | n/a | 3.8574297 | -53.322323 | n/a | n/a | KX356040 |  |  |  | Kocher et al. unpublished |
| *Th. ininii* | n/a | n/a | French Guiana | n/a | n/a | n/a | n/a | M | n/a |  | KJ995653 |  |  | Zapata et al. 2023 |
| *Th. ininii* | ubiq38RO | n/a | Brazil | Rondônia | Itapuã D`Oeste | n/a | n/a | M | n/a | OQ922854 |  |  |  | Costa et al. unpublished |
| *Th. iorlandobaratai* | MT01 | 92309 | Brazil | Pará | Novo Progresso | -7.000549 | -55.406465 | M | Apr-2024 | PV624940 | PV628417 |  | PV651731 | This study |
| *Th. iorlandobaratai* | MT02 | 92310 | Brazil | Pará | Novo Progresso | -7.000549 | -55.406465 | M | Apr-2024 | PV624941 | PV628418 |  | PV651732 | This study |
| *Th. iorlandobaratai* | MT03 | 92311 | Brazil | Pará | Novo Progresso | -7.000549 | -55.406465 | M | Apr-2024 | PV624942 |  |  |  | This study |
| *Th. iorlandobaratai* | MT08 | 92312 | Brazil | Pará | Novo Progresso | -7.000549 | -55.406465 | M | Apr-2024 | PV624945 |  |  |  | This study |
| *Th. iorlandobaratai* | MT10 | 92313 | Brazil | Pará | Novo Progresso | -7.000549 | -55.406465 | M | Apr-2024 | PV624946 |  |  |  | This study |
| *Th. iorlandobaratai* | MT14 | 92314 | Brazil | Pará | Novo Progresso | -7.000549 | -55.406465 | M | Apr-2024 | PV624947 |  |  |  | This study |
| *Th. jariensis* | JRI08 | 92315 | Brazil | Amapá | Laranjal do Jari | -0.657139 | -52.507389 | F | 13–Jan-2023 | PP213816 |  |  |  | Cavalcante et al. 2024 |
| *Th. jariensis* | JRI11 | E-16440 | Brazil | Amapá | Laranjal do Jari | -0.657139 | -52.507389 | M | 13–Jan-2023 | PP213815 |  |  |  | Cavalcante et al. 2024 |
| *Th. jariensis* | JRI12 | 92316 | Brazil | Amapá | Laranjal do Jari | -0.657139 | -52.507389 | F | 13–Jan-2023 | PP213814 |  |  |  | Cavalcante et al. 2024 |
| *Th. jariensis* | JRI14 | 92317 | Brazil | Amapá | Laranjal do Jari | -0.657139 | -52.507389 | M | 13-Jan-2023 |  | PV628416 | PV636962 |  | This study |
| *Th. jariensis* | JRI16 | 92318 | Brazil | Amapá | Laranjal do Jari | -0.657139 | -52.507389 | F | 13–Jan-2023 | PP213813 |  |  |  | Cavalcante et al. 2024 |
| *Th. jariensis* | JRI18 | 92319 | Brazil | Amapá | Laranjal do Jari | -0.657139 | -52.507389 | F | 13–Jan-2023 | PP213812 |  |  |  | Cavalcante et al. 2024 |
| *Th. loretonensis* | ubiq36RORO | n/a | Brazil | Rondônia | Itapuã D`Oeste | n/a | n/a | M | n/a | OQ922855 |  |  |  | Costa et al. unpublished |
| *Th. melloi* | AC336 | 92320 | Brazil | Acre | Rio Branco | -10.072602 | -67.627792 | M | 10-Dec-2022 | PV624893 |  |  |  | This study |
| *Th. melloi* | AC355 | 92321 | Brazil | Acre | Rio Branco | -10.072602 | -67.627792 | M | 10-Dec-2022 | PV624895 |  |  |  | This study |
| *Th. melloi* | AC358 | 92322 | Brazil | Acre | Rio Branco | -10.072602 | -67.627792 | M | 10-Dec-2022 | PV624896 |  |  |  | This study |
| *Th. melloi* | AC359 | 92323 | Brazil | Acre | Rio Branco | -10.072602 | -67.627792 | M | 10-Dec-2022 | PV624897 |  |  |  | This study |
| *Th. melloi* | AM05 | 92324 | Brazil | Amazonas | Labrea | -7.664059 | -65.069582 | M | Feb-2014 | PV624901 |  |  |  | This study |
| *Th. melloi* | AM07 | 92325 | Brazil | Amazonas | Labrea | -7.664059 | -65.069582 | M | Feb-2014 | PV624903 |  |  |  | This study |
| *Th. melloi* | ROB45 | 92326 | Brazil | Amazonas | Canutama | -7.874846 | -63.872774 | M | 01-Oct-2023 | PV625025 |  | PV636974 |  | This study |
| *Th. melloi* | ROB84 | 92327 | Brazil | Rondônia | Candeias do Jamari | -8.954694 | -63.173316 | M | 15-Dec-2023 | PV625031 |  |  | PV651749 | This study |
| *Th. melloi* | ROB85 | 92328 | Brazil | Rondônia | Candeias do Jamari | -8.954694 | -63.173316 | M | 15-Dec-2023 | PV625032 |  | PV636975 | PV651750 | This study |
| *Th. octavioi* | 4R42M37 | 91245a | Brazil | Acre | Brasiléia | -10.945556 | -68.703889 | M | n/a | OP346842 |  |  |  | Pinto et al. 2023 |
| *Th. octavioi* | 4R42M47 | 91258b | Brazil | Acre | Brasiléia | -10.945556 | -68.703889 | M | n/a | OP346843 |  |  |  | Pinto et al. 2023 |
| *Th. octavioi* | 4R43M15 | 91246c | Brazil | Acre | Brasiléia | -10.945556 | -68.703889 | M | n/a | OP346841 |  |  |  | Pinto et al. 2023 |
| *Th. octavioi* | AC314 | 92329 | Brazil | Acre | Xapuri | -10.838528 | -68.377111 | M | 23-Apr-2021 | PV624884 | PV628406 | PV636953 | PV651723 | This study |
| *Th. octavioi* | AC316 | 92330 | Brazil | Acre | Xapuri | -10.838528 | -68.377111 | M | 23-Apr-2021 | PV624885 | PV628407 | PV636954 |  | This study |
| *Th. octavioi* | AC319 | 92331 | Brazil | Acre | Xapuri | -10.838529 | -68.377112 | M | 23-Apr-2021 | PV624886 |  | PV636955 |  | This study |
| *Th. octavioi* | AC320 | 92332 | Brazil | Acre | Xapuri | -10.838528 | -68.377111 | M | 23-Apr-2021 | PV624887 |  |  |  | This study |
| *Th. octavioi* | AC331 | 92333 | Brazil | Acre | Rio Branco | -10.072602 | -67.627792 | M | 10-Dec-2022 | PV624890 |  |  |  | This study |
| *Th. octavioi* | AC335 | 92334 | Brazil | Acre | Rio Branco | -10.072602 | -67.627792 | M | 10-Dec-2022 | PV624892 |  |  |  | This study |
| *Th. octavioi* | AC353 | 92335 | Brazil | Acre | Rio Branco | -10.072602 | -67.627792 | M | 10-Dec-2022 | PV624894 |  |  |  | This study |
| *Th. octavioi* | AC360 | 92336 | Brazil | Acre | Rio Branco | -10.072602 | -67.627792 | M | 10-Dec-2022 | PV624898 |  |  |  | This study |
| *Th. octavioi* | AC361 | 92337 | Brazil | Acre | Rio Branco | -10.072602 | -67.627792 | M | 10-Dec-2022 | PV624899 |  |  |  | This study |
| *Th. pabloi* | n/a | n/a | Ecuador | n/a | n/a | n/a | n/a | M | n/a |  | KJ995655 |  |  | Zapata et al. 2023 |
| *Th. peixotoi* | PNA12B | E-16400 | Brazil | Pará | Itaituba | -4.472778 | -56.286694 | M | 10-Sep-2022 | OQ843042 |  |  |  | Rodrigues et al. 2023 |
| *Th. peixotoi* | PNA13B | E-16401 | Brazil | Pará | Itaituba | -4.472778 | -56.286694 | M | 10-Sep-2022 | OQ843041 |  |  |  | Rodrigues et al. 2023 |
| *Th. peixotoi* | PNA23B | E-16396 | Brazil | Pará | Itaituba | -4.611528 | -56.390361 | M | 12-Nov-2022 | OQ843040 |  |  |  | Rodrigues et al. 2023 |
| *Th. peixotoi* | PNA24b | E-16397 | Brazil | Pará | Itaituba | -4.472778 | -56.286694 | M | 10-Sep-2022 |  |  | OR271585 |  | Rodrigues et al. 2023 |
| *Th. peixotoi* | PNA30B | E-16398 | Brazil | Pará | Itaituba | -4.611528 | -56.390361 | M | 12-Nov-2022 | OQ843038 |  |  |  | Rodrigues et al. 2023 |
| *Th. peixotoi* | PNA45b | 92338 | Brazil | Pará | Itaituba | -4.472778 | -56.286694 | F | 10-Sep-2022 | PV625005 |  |  |  | This study |
| *Th. peixotoi* | PNA52b | 92339 | Brazil | Pará | Itaituba | -4.472778 | -56.286694 | F | 10-Sep-2022 | PV625012 |  |  |  | This study |
| *Th. peixotoi* | PNA76b | 92340 | Brazil | Pará | Itaituba | -4.472778 | -56.286694 | M | 10-Sep-2022 |  | PV628430 | PV636971 | PV651743 | This study |
| *Th. readyi* | PNA10b | 92341 | Brazil | Pará | Itaituba | -4.472778 | -56.286694 | M | 10-Sep-2022 | PV624989 |  |  |  | This study |
| *Th. readyi* | PNA111b | 92342 | Brazil | Pará | Itaituba | -4.472778 | -56.286694 | F | 27-Jan-2023 | PV624994 |  |  |  | This study |
| *Th. readyi* | PNA114b | 92343 | Brazil | Pará | Itaituba | -4.472778 | -56.286694 | F | 27-Jan-2023 | PV624995 |  |  |  | This study |
| *Th. readyi* | PNA124b | 92344 | Brazil | Pará | Itaituba | -4.472778 | -56.286694 | F | 27-Jan-2023 | PV624996 |  |  |  | This study |
| *Th. readyi* | PNA125b | 92345 | Brazil | Pará | Itaituba | -4.472778 | -56.286694 | F | 27-Jan-2023 | PV624997 |  |  |  | This study |
| *Th. readyi* | PNA14b | 92346 | Brazil | Pará | Itaituba | -4.472778 | -56.286694 | M | 10-Sep-2022 | PV624990 |  |  |  | This study |
| *Th. readyi* | PNA15b | 92347 | Brazil | Pará | Itaituba | -4.472778 | -56.286694 | M | 10-Sep-2022 | PV624991 |  |  |  | This study |
| *Th. readyi* | PNA39b | 92348 | Brazil | Pará | Itaituba | -4.472778 | -56.286694 | F | 10-Sep-2022 | PV625000 |  |  |  | This study |
| *Th. readyi* | PNA40b | 92349 | Brazil | Pará | Itaituba | -4.472778 | -56.286694 | F | 10-Sep-2022 | PV625001 |  |  |  | This study |
| *Th. readyi* | PNA41b | 92350 | Brazil | Pará | Itaituba | -4.472778 | -56.286694 | M | 10-Sep-2022 | PV625002 |  |  |  | This study |
| *Th. readyi* | PNA42b | 92351 | Brazil | Pará | Itaituba | -4.472778 | -56.286694 | F | 10-Sep-2022 | PV625003 |  |  |  | This study |
| *Th. readyi* | PNA43b | 92352 | Brazil | Pará | Itaituba | -4.472778 | -56.286694 | F | 10-Sep-2022 | PV625004 |  |  |  | This study |
| *Th. readyi* | PNA46b | 92353 | Brazil | Pará | Itaituba | -4.472778 | -56.286694 | F | 10-Sep-2022 | PV625006 |  |  |  | This study |
| *Th. readyi* | PNA47b | 92354 | Brazil | Pará | Itaituba | -4.472778 | -56.286694 | F | 10-Sep-2022 | PV625007 |  |  |  | This study |
| *Th. readyi* | PNA48b | 92355 | Brazil | Pará | Itaituba | -4.472778 | -56.286694 | M | 10-Sep-2022 | PV625008 |  |  | PV651742 | This study |
| *Th. readyi* | PNA49b | 92356 | Brazil | Pará | Itaituba | -4.472778 | -56.286694 | M | 10-Sep-2022 | PV625009 |  | PV636969 |  | This study |
| *Th. readyi* | PNA50b | 92357 | Brazil | Pará | Itaituba | -4.472778 | -56.286694 | M | 10-Sep-2022 | PV625010 |  | PV636970 |  | This study |
| *Th. readyi* | PNA51b | 92358 | Brazil | Pará | Itaituba | -4.472778 | -56.286694 | F | 10-Sep-2022 | PV625011 |  |  |  | This study |
| *Th. readyi* | PNA56b | 92359 | Brazil | Pará | Itaituba | -4.472778 | -56.286694 | M | 10-Sep-2022 | PV625013 |  |  |  | This study |
| *Th. readyi* | PNA62b | 92360 | Brazil | Pará | Itaituba | -4.472778 | -56.286694 | M | 10-Sep-2022 | PV625014 |  |  |  | This study |
| *Th. ruii* | AM09 | 92361 | Brazil | Amazonas | Labrea | -7.664059 | -65.069582 | M | Feb-2014 | PV624905 | PV628410 |  |  | This study |
| *Th. ruii* | AM10 | 92362 | Brazil | Amazonas | Labrea | -7.664059 | -65.069582 | M | Feb-2014 | PV624906 | PV628411 | PV636957 | PV651725 | This study |
| *Th. sinuosa* | n/a | n/a | Ecuador | n/a | n/a | n/a | n/a | M | n/a |  | KJ995657 |  |  | Zapata et al. 2023 |
| *Th.* sp. | AC325 | 92363 | Brazil | Acre | Rio Branco | -10.072602 | -67.627792 | F | 20-Mar-2024 | PV624888 |  |  |  | This study |
| *Th.* sp. | AC326 | 92364 | Brazil | Acre | Rio Branco | -10.072602 | -67.627792 | F | 20-Mar-2024 | PV624889 |  |  |  | This study |
| *Th.* sp. | PAB13 | 92365 | Brazil | Pará | Belterra | -2.880978 | -54.932743 | F | 28-Jan-2019 | PV624983 |  |  |  | This study |
| *Th.* sp. | PAB15 | 92366 | Brazil | Pará | Belterra | -2.880978 | -54.932743 | F | 28-Jan-2019 | PV624984 |  |  |  | This study |
| *Th.* sp. | PNA102b | 92367 | Brazil | Pará | Itaituba | -4.472778 | -56.286694 | F | 27-Jan-2023 | PV624992 |  |  |  | This study |
| *Th.* sp. | PNA107b | 92368 | Brazil | Pará | Itaituba | -4.472778 | -56.286694 | F | 27-Jan-2023 | PV624993 |  |  |  | This study |
| *Th.* sp. | ROB47 | 92369 | Brazil | Amazonas | Canutama | -7.874846 | -63.872774 | F | 01-Oct-2023 | PV625026 |  |  |  | This study |
| *Th.* sp. of Amapá | APC01 | n/a | Brazil | Amapá | Porto Grande | 0.574187 | -51.253091 | M | 26-Apr-2024 | PV624923 |  |  |  | This study |
| *Th.* sp. of Amapá | APC04 | n/a | Brazil | Amapá | Porto Grande | 0.574187 | -51.253091 | M | 26-Apr-2024 | PV624924 |  | PV636958 |  | This study |
| *Th.* sp. of Amapá | APC05 | 92370 | Brazil | Amapá | Porto Grande | 0.574187 | -51.253091 | M | 26-Apr-2024 | PV624925 |  |  |  | This study |
| *Th.* sp. of Amapá | APC06 | n/a | Brazil | Amapá | Porto Grande | 0.574187 | -51.253091 | M | 26-Apr-2024 | PV624926 |  |  | PV651728 | This study |
| *Th.* sp. of Amapá | APC08 | 92371 | Brazil | Amapá | Porto Grande | 0.574187 | -51.253091 | F | 26-Apr-2024 | PV624927 |  |  |  | This study |
| *Th.* sp. of Amapá | APC09 | n/a | Brazil | Amapá | Porto Grande | 0.574187 | -51.253091 | F | 26-Apr-2024 | PV624928 |  |  |  | This study |
| *Th.* sp. of Amapá | APC10 | n/a | Brazil | Amapá | Porto Grande | 0.574187 | -51.253091 | M | 26-Apr-2024 | PV624929 |  |  |  | This study |
| *Th.* sp. of Amapá | APC11 | n/a | Brazil | Amapá | Porto Grande | 0.574187 | -51.253091 | F | 26-Apr-2024 | PV624930 |  |  | PV651729 | This study |
| *Th.* sp. of Amapá | APC13 | n/a | Brazil | Amapá | Porto Grande | 0.574187 | -51.253091 | F | 26-Apr-2024 | PV624931 |  |  |  | This study |
| *Th.* sp. of Amapá | APC20 | n/a | Brazil | Amapá | Porto Grande | 0.574187 | -51.253091 | M | 26-Apr-2024 | PV624932 |  |  |  | This study |
| *Th. ubiquitalis* | AC332 | 92372 | Brazil | Acre | Rio Branco | -10.072602 | -67.627792 | M | 10-Dec-2022 | PV624891 |  |  |  | This study |
| *Th. ubiquitalis* | AM06 | 92373 | Brazil | Amazonas | Labrea | -7.664059 | -65.069582 | M | Feb-2014 | PV624902 |  |  |  | This study |
| *Th. ubiquitalis* | APB03 | 92374 | Brazil | Amapá | Calcoene | 2.318833 | -51.653402 | M | 30-Nov-2021 | PV624908 |  |  |  | This study |
| *Th. ubiquitalis* | APB05 | 92375 | Brazil | Amapá | Calcoene | 2.318833 | -51.653402 | M | 30-Nov-2021 | PV624910 |  |  |  | This study |
| *Th. ubiquitalis* | APB07 | 92376 | Brazil | Amapá | Calcoene | 2.318833 | -51.653402 | M | 30-Nov-2021 | PV624912 |  |  |  | This study |
| *Th. ubiquitalis* | MT04 | 92377 | Brazil | Pará | Novo Progresso | -7.000549 | -55.406465 | M | Apr-2024 | PV624943 |  |  |  | This study |
| *Th. ubiquitalis* | MT05 | 92378 | Brazil | Pará | Novo Progresso | -7.000549 | -55.406465 | M | Apr-2024 | PV624944 |  |  |  | This study |
| *Th. ubiquitalis* | PA04 | 92379 | Brazil | Pará | Belém | -1.430208 | -48.456568 | M | 13-Mar-2023 | PV624956 |  |  |  | This study |
| *Th. ubiquitalis* | PA05 | 92380 | Brazil | Pará | Belém | -1.430208 | -48.456568 | M | 13-Mar-2023 | PV624957 |  |  |  | This study |
| *Th. ubiquitalis* | PA06 | 92381 | Brazil | Pará | Belém | -1.430208 | -48.456568 | M | 13-Mar-2023 | PV624958 |  |  |  | This study |
| *Th. ubiquitalis* | PNA17b | 92382 | Brazil | Pará | Itaituba | -4.472778 | -56.286694 | M | 10-Sep-2022 | PV624998 |  |  |  | This study |
| *Th. ubiquitalis* | PNA28b | 92383 | Brazil | Pará | Itaituba | -4.611528 | -56.390361 | M | 12-Nov-2022 | PV624999 |  |  |  | This study |
| *Th. ubiquitalis* | ROB49 | 92384 | Brazil | Rondônia | Candeias do Jamari | -8.954694 | -63.173316 | M | 15-Dec-2023 | PV625027 | PV628433 |  | PV651746 | This study |
| *Th. ubiquitalis* | ROB50 | 92385 | Brazil | Rondônia | Candeias do Jamari | -8.954694 | -63.173316 | M | 15-Dec-2023 | PV625028 | PV628434 |  | PV651747 | This study |
| *Th. ubiquitalis* | ROB60 | 92386 | Brazil | Rondônia | Candeias do Jamari | -8.954694 | -63.173316 | M | 15-Dec-2023 | PV625030 |  |  | PV651748 | This study |
| *Th. ubiquitalis* | Ubiq103ROpotosi | n/a | Brazil | Rondônia | Itapuã D`Oeste | n/a | n/a | M | n/a | OQ922861 |  |  |  | Costa et al. unpublished |
| *Th. ubiquitalis* | ubiq105ROSM | n/a | Brazil | Rondônia | Itapuã D`Oeste | n/a | n/a | M | n/a | OQ922856 |  |  |  | Costa et al. unpublished |
| *Th. ubiquitalis* | ubiq108ROSM | n/a | Brazil | Rondônia | Itapuã D`Oeste | n/a | n/a | M | n/a | OQ922860 |  |  |  | Costa et al. unpublished |
| *Th. ubiquitalis* | ubiq114ROSM | n/a | Brazil | Rondônia | Itapuã D`Oeste | n/a | n/a | M | n/a | OQ922858 |  |  |  | Costa et al. unpublished |
| *Th. ubiquitalis* | ubiq94ROpotosi | n/a | Brazil | Rondônia | Itapuã D`Oeste | n/a | n/a | M | n/a | OQ922862 |  |  |  | Costa et al. unpublished |
| *Th. velascoi* | n/a | n/a | Ecuador | n/a | n/a | n/a | n/a | M | n/a |  | KJ995658 |  |  | Zapata et al. 2023 |
| *Th. velezbernali* | AMA2.1 | n/a | Colombia | Amazonas | Puerto Nariño | -3.770278 | -70.383056 | M | n/a | OP964359 |  |  |  | Posada-López et al. 2023 |
| *Th. velezbernali* | AMA3.1 | n/a | Colombia | Amazonas | Puerto Nariño | -3.770278 | -70.383056 | M | n/a | OP964358 |  |  |  | Posada-López et al. 2023 |
| *Th. velezbernali* | AMCT8.2 | n/a | Colombia | Amazonas | Puerto Nariño | -3.770278 | -70.383056 | M | n/a | OP964360 |  |  |  | Posada-López et al. 2023 |
| *Th. viannamartinsi* | ESB01 | 92387 | Brazil | Bahia | Porto Seguro | -20.214722 | -41.041944 | M | 14-Nov-2019 | PV624933 |  |  |  | This study |
| *Th. viannamartinsi* | ESB02 | 92388 | Brazil | Bahia | Porto Seguro | -20.214722 | -41.041944 | M | 14-Nov-2019 | PV624934 |  |  |  | This study |
| *Th. viannamartinsi* | ESB03 | 92389 | Brazil | Bahia | Porto Seguro | -20.214722 | -41.041944 | M | 14-Nov-2019 | PV624935 |  |  |  | This study |
| *Th. viannamartinsi* | ESB04 | 92390 | Brazil | Bahia | Porto Seguro | -20.214722 | -41.041944 | M | 14-Nov-2019 | PV624936 |  |  |  | This study |
| *Th. viannamartinsi* | ESB05 | 92391 | Brazil | Bahia | Porto Seguro | -20.214722 | -41.041944 | M | 14-Nov-2019 | PV624937 | PV628414 | PV636959 |  | This study |
| *Th. viannamartinsi* | ESB06 | 92392 | Brazil | Bahia | Porto Seguro | -20.214722 | -41.041944 | M | 14-Nov-2019 | PV624938 | PV628415 | PV636960 | PV651730 | This study |
| *Th. viannamartinsi* | ESB07 | 92393 | Brazil | Bahia | Porto Seguro | -20.214722 | -41.041944 | M | 14-Nov-2019 | PV624939 |  | PV636961 |  | This study |
| *Th. viannamartinsi* | LBMI#0375 | 90751 | Brazil | Bahia | Wenceslau Guimarães | -13.584444 | -39.708889 | M | n/a | KP113048 |  |  |  | Pinto et al. 2015 |
| *Th. viannamartinsi* | LBMI#0376 | 90752 | Brazil | Bahia | Wenceslau Guimarães | -13.584444 | -39.708889 | M | n/a | KP113047 |  |  |  | Pinto et al. 2015 |
| *Th. viannamartinsi* | LBMI#0377 | 90753 | Brazil | Bahia | Wenceslau Guimarães | -13.584444 | -39.708889 | M | n/a | KP113049 |  |  |  | Pinto et al. 2015 |
| *Th. viannamartinsi* | LBMI#0379 | 90755 | Brazil | Bahia | Wenceslau Guimarães | -13.584444 | -39.708889 | M | n/a | KP113050 |  |  |  | Pinto et al. 2015 |
| *Th. viannamartinsi* | LBMI#0380 | 90756 | Brazil | Bahia | Wenceslau Guimarães | -13.584444 | -39.708889 | F | n/a | KP113051 |  |  |  | Pinto et al. 2015 |
| *Th. viannamartinsi* | MUC06 | 92394 | Brazil | Alagoas | Murici | -9.187099 | -35.924358 | M | 27-Dec-2022 | PV624948 | PV628419 | OR271587 | PV651733 | This study; Rodrigues et al. 2023 |
| *Th. viannamartinsi* | MUC07 | 92395 | Brazil | Alagoas | Murici | -9.187099 | -35.924358 | M | 27-Dec-2022 | PV624949 | PV628420 | OR271586 |  | This study; Rodrigues et al. 2023 |
| *Th. viannamartinsi* | MUC10 | 92396 | Brazil | Alagoas | Murici | -9.187099 | -35.924358 | F | 27-Dec-2022 | PV624950 |  |  |  | This study |
| *Th. viannamartinsi* | MUC12 | 92397 | Brazil | Alagoas | Murici | -9.187099 | -35.924358 | F | 27-Dec-2022 | PV624951 |  |  |  | This study |
| *Th. viannamartinsi* | MUC13 | 92398 | Brazil | Alagoas | Murici | -9.187099 | -35.924358 | F | 27-Dec-2022 | PV624952 |  |  |  | This study |
| *Th. viannamartinsi* | MUC22 | 92399 | Brazil | Alagoas | Murici | -9.187099 | -35.924358 | M | 27-Dec-2022 | PV624953 |  |  |  | This study |
| *Th. viannamartinsi* | MUC23 | 92400 | Brazil | Alagoas | Murici | -9.187099 | -35.924358 | M | 27-Dec-2022 | PV624954 |  |  |  | This study |
| *Th. viannamartinsi* | MUC24 | 92401 | Brazil | Alagoas | Murici | -9.187099 | -35.924358 | M | 27-Dec-2022 | PV624955 |  |  |  | This study |
| *Th. viannamartinsi* | PPB01 | 92402 | Brazil | Bahia | Porto Seguro | -16.466086 | -39.289831 | M | 14-Nov-2019 | PV625015 |  |  |  | This study |
| *Th. viannamartinsi* | PPB02 | 92403 | Brazil | Bahia | Porto Seguro | -16.466086 | -39.289831 | M | 14-Nov-2019 | PV625016 |  |  |  | This study |
| *Th. viannamartinsi* | PPB03 | 92404 | Brazil | Bahia | Porto Seguro | -16.466086 | -39.289831 | M | 14-Nov-2019 | PV625017 |  |  |  | This study |
| *Th. viannamartinsi* | PPB05 | 92405 | Brazil | Bahia | Porto Seguro | -16.466086 | -39.289831 | M | 14-Nov-2019 | PV625018 |  |  |  | This study |
| *Th. viannamartinsi* | PPB07 | 92406 | Brazil | Bahia | Porto Seguro | -16.466086 | -39.289831 | M | 14-Nov-2019 | PV625019 |  |  |  | This study |
| *Re. reburrus* | n/a | n/a | Colombia | Chocó | Bahía Solano | 6.3717 | -77.3695 | n/a | 17-May-2011 | KC921282 |  |  |  | Contreras Gutierrez et al. 2014 |
| *Re. reburrus* | n/a | n/a | Colombia | Chocó | Bahía Solano | 6.3717 | -77.3695 | n/a | 17-May-2011 | KC921283 |  |  |  | Contreras Gutierrez et al. 2014 |
| *Re. reburrus* | n/a | n/a | Colombia | Chocó | Bahía Solano | 6.3717 | -77.3695 | n/a | 17-May-2011 | KC921284 |  |  |  | Contreras Gutierrez et al. 2014 |
| *Re. reburrus* | n/a | n/a | Ecuador | n/a | n/a | n/a | n/a | F | n/a |  | KJ995656 |  |  | Zapata et al. 2023 |
| *Re. reburrus* | n/a | n/a | Ecuador | Cañar | Ocaña | n/a | n/a | n/a | n/a |  |  | AB479930 |  | Kuwahara et al. 2009 |
| *Sh. richardwardi* | 3605FRO | n/a | Brazil | Rondônia | Itapuã D`Oeste | n/a | n/a | F | n/a | OQ922481 |  |  |  | Costa et al. unpublished |
| *Sh. richardwardi* | 3628FRO | n/a | Brazil | Rondônia | Itapuã D`Oeste | n/a | n/a | F | n/a | OQ922480 |  |  |  | Costa et al. unpublished |
| *Sh. richardwardi* | 3739ARO | n/a | Brazil | Rondônia | Itapuã D`Oeste | n/a | n/a | F | n/a | OQ922479 |  |  |  | Costa et al. unpublished |
| *Sh. richardwardi* | 3818FRO | n/a | Brazil | Rondônia | Itapuã D`Oeste | n/a | n/a | F | n/a | OQ922485 |  |  |  | Costa et al. unpublished |
| *Sh. richardwardi* | n/a | n/a | Ecuador | n/a | n/a | n/a | n/a | M | n/a |  | KJ995647 |  |  | Zapata et al. 2023 |
| *Sh. richardwardi* | PAC11 | 92407 | Brazil | Pará | Vitoria do Xingu | -3.416633 | -51.892750 | F | 24-May-2023 | PV624985 | PV628428 |  | PV651740 | This study |
| *Sh. richardwardi* | PAC12 | 92408 | Brazil | Pará | Vitoria do Xingu | -3.416633 | -51.892750 | F | 24-May-2023 | PV624986 | PV628429 |  | PV651741 | This study |
| *Sh. richardwardi* | PAC13 | 92409 | Brazil | Pará | Vitoria do Xingu | -3.416633 | -51.892750 | F | 24-May-2023 | PV624987 |  |  |  | This study |
| *Sh. richardwardi* | PAC14 | 92410 | Brazil | Pará | Vitoria do Xingu | -3.416633 | -51.892750 | F | 24-May-2023 | PV624988 |  |  |  | This study |
| *Sh. shawi* | 3924ARO | n/a | Brazil | Rondônia | Itapuã D`Oeste | n/a | n/a | F | n/a | OQ922491 |  |  |  | Costa et al. unpublished |
| *Sh. shawi* | 395RO | n/a | Brazil | Rondônia | Itapuã D`Oeste | n/a | n/a | M | n/a | OQ922490 |  |  |  | Costa et al. unpublished |
| *Sh. shawi* | 4R43M33 | 91250c | Brazil | Acre | Brasiléia | -10.945556 | -68.703889 | M | n/a | OP346795 |  |  |  | Pinto et al. 2023 |
| *Sh. shawi* | 884JRO | n/a | Brazil | Rondônia | Itapuã D`Oeste | n/a | n/a | F | n/a | OQ922492 |  |  |  | Costa et al. unpublished |
| *Ny. anduzei* | CAR363 | n/a | Brazil | Maranhão | Governador Newton Bello | -3.445417 | -46.255556 | M | 18-Jan-2023 | OR555658 |  |  |  | Rodrigues & Galati 2024 |
| *Ny. anduzei* | CAR375 | n/a | Brazil | Maranhão | Governador Newton Bello | -3.445417 | -46.255556 | F | 18-Jan-2023 | OR555659 |  |  |  | Rodrigues & Galati 2024 |
| *Ny. anduzei* | CAR95 | n/a | Brazil | Maranhão | Governador Newton Bello | -3.445417 | -46.255556 | F | 18-Jan-2023 | OR555657 |  |  |  | Rodrigues & Galati 2024 |
| *Ny. antunesi* | CAR207 | n/a | Brazil | Maranhão | Governador Newton Bello | -3.445417 | -46.255556 | F | 18-Jan-2023 | OR555660 |  |  |  | Rodrigues & Galati 2024 |
| *Ny. antunesi* | CAR249 | n/a | Brazil | Maranhão | Governador Newton Bello | -3.445417 | -46.255556 | F | 18-Jan-2023 | OR555667 |  |  |  | Rodrigues & Galati 2024 |
| *Ny. antunesi* | CAR252 | n/a | Brazil | Maranhão | Governador Newton Bello | -3.445417 | -46.255556 | M | 18-Jan-2023 | OR555666 |  |  |  | Rodrigues & Galati 2024 |
| *Ny. antunesi* | CAR255 | n/a | Brazil | Maranhão | Governador Newton Bello | -3.445417 | -46.255556 | F | 18-Jan-2023 | OR555665 |  |  |  | Rodrigues & Galati 2024 |
| *Ny. antunesi* | n/a | n/a | Ecuador | n/a | n/a | n/a | n/a | M | n/a |  | KJ995644 |  |  | Zapata et al. 2023 |
| *Ny. delsionatali* | 103MRO | n/a | Brazil | Rondônia | Pimenta Bueno | n/a | n/a | M | n/a | OQ922473 |  |  |  | Costa et al. unpublished |
| *Ny. delsionatali* | 107MRO | n/a | Brazil | Rondônia | Pimenta Bueno | n/a | n/a | M | n/a | OQ922471 |  |  |  | Costa et al. unpublished |
| *Ny. delsionatali* | 443MRO | n/a | Brazil | Rondônia | Pimenta Bueno | n/a | n/a | M | n/a | OQ922472 |  |  |  | Costa et al. unpublished |
| *Ny. fraihai* | AMA57.1 | n/a | Colombia | Amazonas | Puerto Nariño | -3.770278 | -70.383056 | F | n/a | OP964270 |  |  |  | Posada-López et al. 2023 |
| *Ny. fraihai* | AMA81.2 | n/a | Colombia | Amazonas | Leticia | -4.208056 | -69.943056 | F | n/a | OP964271 |  |  |  | Posada-López et al. 2023 |
| *Ny. fraihai* | AMCK11-1.3 | n/a | Colombia | Amazonas | Leticia | -4.208056 | -69.943056 | F | n/a | OP964268 |  |  |  | Posada-López et al. 2023 |
| *Ny. fraihai* | AMCK11-15.2 | n/a | Colombia | Amazonas | Leticia | -4.208056 | -69.943056 | F | n/a | OP964272 |  |  |  | Posada-López et al. 2023 |
| *Ny. fraihai* | AMCK11-17.2 | n/a | Colombia | Amazonas | Leticia | -4.208056 | -69.943056 | F | n/a | OP964269 |  |  |  | Posada-López et al. 2023 |
| *Ny. intermedia* | LBMI#0217 | 90340 | Brazil | Espirito Santo | Pancas | -19.228 | -40.758 | M | 07-Feb-2011 | KP112719 |  |  |  | Pinto et al. 2015 |
| *Ny. intermedia* | LBMI#0218 | 90341 | Brazil | Espirito Santo | Pancas | -19.228 | -40.758 | M | 07-Feb-2011 | KP112718 |  |  |  | Pinto et al. 2015 |
| *Ny. intermedia* | LBMI#0219 | 90342 | Brazil | Espirito Santo | Pancas | -19.228 | -40.758 | M | 07-Feb-2011 | KP112716 |  |  |  | Pinto et al. 2015 |
| *Ny. intermedia* | LBMI#0220 | 90343 | Brazil | Espirito Santo | Pancas | -19.228 | -40.758 | M | 07-Feb-2011 | KP112715 |  |  |  | Pinto et al. 2015 |
| *Ny. intermedia* | LBMI#0254 | 90411 | Brazil | Espirito Santo | Pancas | -19.228 | -40.758 | F | 24-May-2011 | KP112717 |  |  |  | Pinto et al. 2015 |
| *Ny. intermedia* | n/a | n/a | Brazil | n/a | n/a | n/a | n/a | M | n/a |  | KJ995645 |  |  | Zapata et al. 2023 |
| *Ny. neivai* | n/a | n/a | Brazil | Santa Catarina | Tubarão | -28.520178 | -49.017525 | n/a | 25-Jun-2019 | OP719772 |  |  |  | Cardoso et al. 2024 |
| *Ny. neivai* | n/a | n/a | Brazil | Santa Catarina | Tubarão | -28.520178 | -49.017525 | n/a | 25-Jun-2019 | OP719774 |  |  |  | Cardoso et al. 2024 |
| *Ny. neivai* | n/a | n/a | Brazil | n/a | n/a | n/a | n/a | M | n/a |  | KJ995646 |  |  | Zapata et al. 2023 |
| *Ny. trapidoi* | n/a | n/a | Colombia | Caldas | Victoria | 5.3333 | -74.9333 | n/a | 29-Jul-2010 | KC921299 |  |  |  | Contreras Gutierrez et al. 2014 |
| *Ny. trapidoi* | n/a | n/a | Colombia | Caldas | Victoria | 5.3333 | -74.9333 | n/a | 30-Jul-2010 | KC921300 |  |  |  | Contreras Gutierrez et al. 2014 |
| *Ny. trapidoi* | n/a | n/a | Colombia | Caldas | Victoria | 5.3333 | -74.9333 | n/a | 31-Jul-2010 | KC921301 |  |  |  | Contreras Gutierrez et al. 2014 |
| *Ny. trapidoi* | n/a | n/a | Colombia | Caldas | Victoria | 5.3333 | -74.9333 | n/a | 31-Jul-2010 | KC921302 |  |  |  | Contreras Gutierrez et al. 2014 |
| *Ny. trapidoi* | n/a | n/a | Colombia | Caldas | Victoria | 5.3333 | -74.9333 | n/a | 30-Jul-2010 | KC921303 |  |  |  | Contreras Gutierrez et al. 2014 |
| *Ny. trapidoi* | n/a | n/a | Ecuador | n/a | n/a | n/a | n/a | F | n/a |  | KJ995648 |  |  | Zapata et al. 2023 |
| *Ny. umbratilis* | n/a | n/a | Brazil | n/a | n/a | n/a | n/a | n/a | n/a | KF467552 |  |  |  | Scarpassa and Alencar 2013 |
| *Ny. umbratilis* | n/a | n/a | Brazil | n/a | n/a | n/a | n/a | n/a | n/a | KF467553 |  |  |  | Scarpassa and Alencar 2013 |
| *Ny. umbratilis* | n/a | n/a | Brazil | n/a | n/a | n/a | n/a | n/a | n/a | KF467554 |  |  |  | Scarpassa and Alencar 2013 |
| *Ny. umbratilis* | n/a | n/a | Brazil | n/a | n/a | n/a | n/a | n/a | n/a | KF467555 |  |  |  | Scarpassa and Alencar 2013 |
| *Ny. umbratilis* | n/a | n/a | Brazil | n/a | n/a | n/a | n/a | n/a | n/a | KF467556 |  |  |  | Scarpassa and Alencar 2013 |
| *Ny. umbratilis* | n/a | n/a | Brazil | n/a | n/a | n/a | n/a | F | n/a |  | KJ995649 |  |  | Zapata et al. 2023 |
| *Ny. urbinattii* | 148MRO | n/a | Brazil | Rondônia | Machadinho d`Oeste | n/a | n/a | M | n/a | OQ922515 |  |  |  | Costa et al. unpublished |
| *Ny. urbinattii* | 158MRO | n/a | Brazil | Rondônia | Machadinho d`Oeste | n/a | n/a | M | n/a | OQ922513 |  |  |  | Costa et al. unpublished |
| *Ny. urbinattii* | 160ERO | n/a | Brazil | Rondônia | Machadinho d`Oeste | n/a | n/a | M | n/a | OQ922518 |  |  |  | Costa et al. unpublished |
| *Ny. urbinattii* | 86MRO | n/a | Brazil | Rondônia | Pimenta Bueno | n/a | n/a | M | n/a | OQ922516 |  |  |  | Costa et al. unpublished |
| *Ny. whitmani* | LBMI#0096 | 90433 | Brazil | Espirito Santo | Iuna | -20.350556 | -41.724167 | M | 16-Aug-2011 | KP112766 |  |  |  | Pinto et al. 2015 |
| *Ny. whitmani* | LBMI#0405 | 90773 | Brazil | Mato Grosso | Caceres | -16.402 | -57.499 | M | 01-Dec-2012 | KP112763 |  |  |  | Pinto et al. 2015 |
| *Ny. whitmani* | LBMI#0407 | 90775 | Brazil | Mato Grosso | Caceres | -16.402 | -57.499 | M | 01-Dec-2012 | KP112765 |  |  |  | Pinto et al. 2015 |
| *Ny. whitmani* | LBMI#0415 | 90783 | Brazil | Mato Grosso | Caceres | -16.402 | -57.499 | M | 01-Dec-2012 | KP112762 |  |  |  | Pinto et al. 2015 |
| *Ny. whitmani* | LBMI#0424 | 90792 | Brazil | Mato Grosso | Caceres | -16.402 | -57.499 | M | 01-Dec-2012 | KP112764 |  |  |  | Pinto et al. 2015 |
| *Ny. whitmani* | n/a | n/a | Brazil | n/a | n/a | n/a | n/a | M | n/a |  | KJ995650 |  |  | Zapata et al. 2023 |
| *Ny. ylephiletor* | COR7.1 | n/a | Costa Rica | Limon | Sibuju | 9.960 | -84.047 | F | n/a | OP964286 |  |  |  | Posada-López et al. 2023 |
| *Ny. ylephiletor* | n/a | n/a | Nicaragua | n/a | n/a | n/a | n/a | M | n/a |  | KJ995651 |  |  | Zapata et al. 2023 |
| *Ny. yuilli pajoti* | AMA5.1 | n/a | Colombia | Amazonas | Puerto Nariño | -3.770278 | -70.383056 | F | n/a | OP964287 |  |  |  | Posada-López et al. 2023 |
| *Ny. yuilli pajoti* | AMCD5.2 | n/a | Colombia | Amazonas | Puerto Nariño | -3.770278 | -70.383056 | F | n/a | OP964288 |  |  |  | Posada-López et al. 2023 |
| *Ny. yuilli pajoti* | AMCT9.1 | n/a | Colombia | Amazonas | Puerto Nariño | -3.770278 | -70.383056 | F | n/a | OP964289 |  |  |  | Posada-López et al. 2023 |
| *Ny. yuilli pajoti* | n/a | n/a | French Guiana | n/a | n/a | n/a | n/a | M | n/a |  | KJ995652 |  |  | Zapata et al. 2023 |
| *Ny. yuilli yuilli* | n/a | n/a | Colombia | Caldas | Victoria | 5.3333 | -74.9333 | n/a | 30-Jul-2010 | KC921319 |  |  |  | Contreras Gutierrez et al. 2014 |
| *Ny. yuilli yuilli* | n/a | n/a | Colombia | Caldas | Victoria | 5.3333 | -74.9333 | n/a | 30-Jul-2010 | KC921320 |  |  |  | Contreras Gutierrez et al. 2014 |
| *Ny. yuilli yuilli* | n/a | n/a | Colombia | Caldas | Victoria | 5.3333 | -74.9333 | n/a | 29-Jul-2010 | KC921321 |  |  |  | Contreras Gutierrez et al. 2014 |
| *Ny. yuilli yuilli* | n/a | n/a | Colombia | Caldas | Victoria | 5.3333 | -74.9333 | n/a | 13-Oct-2010 | KC921322 |  |  |  | Contreras Gutierrez et al. 2014 |
| *Ny. yuilli yuilli* | n/a | n/a | Colombia | Caldas | Victoria | 5.3333 | -74.9333 | n/a | 13-Oct-2010 | KC921323 |  |  |  | Contreras Gutierrez et al. 2014 |
