# Supplementary material for: On the integrative taxonomy of Trichophoromyia Barretto, 1962 and its relationship with Nyssomyia Barretto, 1962 (Diptera, Psychodidae, Phlebotominae): species delimitation, phylogeny, genus and subgenus description
Source: Parasit Vectors. 2025 Dec 19;19:44. doi: 10.1186/s13071-025-07155-6 (PMC12831354; doi:10.1186/s13071-025-07155-6)
Supplement: Supplementary file 3 — Additional File 3: Table S2. List of sand fly species morphologically analyzed in this study. Number of specimens analyzed for each species, locality, and collection in which the specimen can be found. [file 13071_2025_7155_MOESM3_ESM.docx]

**Table S2.** List of sand fly species morphologically analyzed in this study. Number of specimens analyzed for each species, locality, and collection in which the specimen can be found.

| **Genus/Subgenus** | **Species** | ***n* male** | ***n* female** | **Country** | **State/Departament** | **Locality/Municipality** | **Collection** |
| --- | --- | --- | --- | --- | --- | --- | --- |
| *Trichophoromyia* (*Trichophoromyia*) | sp. do Amapá | 10 | 10 | Brazil | Amapá | Porto Grande | HEP/FSP/USP |
|  | *ubiquitalis* | 1 | 1 | Brazil | Acre | Acrelânia | HEP/FSP/USP |
|  |  | 1 | - | Brazil | Acre | Rio Branco | FIOCRUZ/COLFLEB |
|  |  | 3 | - | Brazil | Amapá | Calçoene | FIOCRUZ/COLFLEB |
|  |  | 1 | - | Brazil | Amapá | Laranjal do Jari | HEP/FSP/USP |
|  |  | 1 | - | Brazil | Amapá | S. F. Iratapuru | HEP/FSP/USP |
|  |  | 1 | - | Brazil | Amazonas | Lábrea | FIOCRUZ/COLFLEB |
|  |  | 3 | - | Brazil | Pará | Belém | FIOCRUZ/COLFLEB |
|  |  | 2 | - | Brazil | Pará | Itaituba | FIOCRUZ/COLFLEB |
|  |  | - | 2 | Brazil | Pará | Marabá | HEP/FSP/USP |
|  |  | 1 | - | Brazil | Pará | Melgaço | HEP/FSP/USP |
|  |  | 2 | - | Brazil | Pará | Novo Progresso | FIOCRUZ/COLFLEB |
|  |  | 3 | - | Brazil | Rondônia | Candeias do Jamari | FIOCRUZ/COLFLEB |
|  | *uniniensis ** | 2 | - | Brazil | Amazonas | Parque Nacional do Jaú | FIOCRUZ/COLFLEB |
| *Trichophoromyia* (*Dilermandomyia*) | *adelsonsouzai* | 6 | - | Brazil | Pará | Vitoria do Xingu | FIOCRUZ/COLFLEB |
|  | *auraensis* | 5 | - | Brazil | Acre | Brasiléia | FIOCRUZ/COLFLEB |
|  |  | 1 | - | Brazil | Acre | Rio Branco | FIOCRUZ/COLFLEB |
|  |  | 4 | - | Brazil | Acre | Xapuri | FIOCRUZ/COLFLEB |
|  |  | 2 | - | Brazil | Amazonas | Manaus | FIOCRUZ/COLFLEB |
|  |  | 1 | - | Peru | Junin | Chanchamayo | HEP/FSP/USP |
|  |  | 1 | - | Peru | Madre de Dios | Tambopata | HEP/FSP/USP |
|  |  | 3 | - | Brazil | Maranhão | Governador Newton Bello | FIOCRUZ/COLFLEB |
|  |  | 1 | - | Peru | Cusco | Pilcopata | HEP/FSP/USP |
|  |  | 1 | - | Peru | Puno | Sandia | HEP/FSP/USP |
|  | *brachipyga* | 5 | - | Brazil | Amapá | Pedra Branca do Amapari | FIOCRUZ/COLFLEB |
|  |  | 1 | 1 | French Guyane | Haut Oyapock | n/a | HEP/FSP/USP |
|  |  | 7 | - | Brazil | Pará | Belém | FIOCRUZ/COLFLEB |
|  | *castanheirai* | 7 | - | Brazil | Pará | Belterra | FIOCRUZ/COLFLEB |
|  | *clitella* | 1 | - | Brazil | Rondônia | Candeias do Jamari | FIOCRUZ/COLFLEB |
|  | *eurypyga* | 1 | - | Brazil | Amazonas | Labrea | FIOCRUZ/COLFLEB |
|  |  | 1 | - | Brazil | Amazonas | Manaus | FIOCRUZ/COLFLEB |
|  |  | 4 | - | Brazil | Pará | Belterra | FIOCRUZ/COLFLEB |
|  | *flochi* | 5 | - | Brazil | Amazonas | Canutama | FIOCRUZ/COLFLEB |
|  | *gibba* | 8 | - | Brazil | Amapá | Calçoene | FIOCRUZ/COLFLEB |
|  | *iorlandobaratai* | 6 | - | Brazil | Pará | Novo Progresso | FIOCRUZ/COLFLEB |
|  | *jariensis* | 6 | - | Brazil | Amapá | Laranjal do Jari | FIOCRUZ/COLFLEB |
|  | *macrisae* | 1 | - | Peru | Cusco | n/a | HEP/FSP/USP |
|  |  | 10 | - | Peru | Madre de Dios | n/a | HEP/FSP/USP |
|  | *melloi* | 4 | - | Brazil | Acre | Rio Branco | FIOCRUZ/COLFLEB |
|  |  | 1 | - | Brazil | Amazonas | Canutama | FIOCRUZ/COLFLEB |
|  |  | 2 | - | Brazil | Amazonas | Labrea | FIOCRUZ/COLFLEB |
|  |  | 2 | - | Brazil | Rondônia | Candeias do Jamari | FIOCRUZ/COLFLEB |
|  | *nemorosa* | 2 | - | Peru | Cusco | n/a | HEP/FSP/USP |
|  | *octavioi* | 3 | - | Brazil | Acre | Brasiléia | FIOCRUZ/COLFLEB |
|  |  | 5 | - | Brazil | Acre | Rio Branco | FIOCRUZ/COLFLEB |
|  |  | 4 | - | Brazil | Acre | Xapuri | FIOCRUZ/COLFLEB |
|  | *peixotoi* | 6 | 2 | Brazil | Pará | Itaituba | FIOCRUZ/COLFLEB |
|  | *readyi* | 9 | 11 | Brazil | Pará | Itaituba | FIOCRUZ/COLFLEB |
|  | *ruifreitasi* | 1 | - | Brazil | Acre | Xapuri | HEP/FSP/USP |
|  | *ruii* | 2 | - | Brazil | Amazonas | Labrea | FIOCRUZ/COLFLEB |
|  | *velezbernali* | 5 | 4 | Colombia | Amazonas | Puerto Nariño | HEP/FSP/USP |
|  | *viannamartinsi* | 5 | 3 | Brazil | Alagoas | Murici | FIOCRUZ/COLFLEB |
|  |  | 12 | - | Brazil | Bahia | Porto Seguro | FIOCRUZ/COLFLEB |
|  |  | 4 | 1 | Brazil | Bahia | Wenceslau Guimarães | FIOCRUZ/COLFLEB |
|  | *wilkersoni* | 5 | 7 | Ecuador | Zamora | Corumbatza | HEP/FSP/USP |
| *Reburrus* | *reburrus ** | 1 | 1 | Panama | Darien | Tacarcuna | MZUSP |
|  | *reburrus* | 3 | 2 | Colombia | Valle Del Cauca | Dagua | n/a |
| *Shawmyia* | *richardwardi* |  | 1 | Brazil | Pará | Belém | HEP/FSP/USP |
|  | *richardwardi* |  | 1 | Brazil | Rondônia | Porto Velho | HEP/FSP/USP |
|  | *richardwardi* | 14 | 3 | Brazil | Pará | Marabá | HEP/FSP/USP |
|  | *richardwardi* | 2 |  | Brazil | Roraima | Mucajaí | HEP/FSP/USP |
|  | *richardwardi* |  | 2 | Brazil | Pará | Parque Nacional de Amazônia | HEP/FSP/USP |
|  | *richardwardi* |  | 1 | Brazil | Acre | Xapuri | HEP/FSP/USP |
|  | *richardwardi ** | 2 |  | Brazil | Pará | Altamira | FIOCRUZ/COLFLEB |
|  | *shawi* | 3 | 2 | Brazil | Acre | Xapuri | HEP/FSP/USP |
|  | *shawi ** | 4 |  | Brazil | Pará | Marabá | FIOCRUZ/COLFLEB |

* Analyzed specimens of the species type series
